# Environmental Variability Shapes Life‐History Trade‐Offs Within and Between Populations of a Long‐Lived Seabird

**DOI:** 10.1111/ele.70384

**Published:** 2026-04-16

**Authors:** Bertille Mohring, Jonathan R. Potts, Alastair J. Wilson, Denis Réale, Richard A. Phillips, Henri Weimerskirch, Christophe Barbraud, Ashley Bennison, Karine Delord, Andrew G. Wood, Samuel Peroteau, Etienne Rouby, Francesco Ventura, Samantha C. Patrick

**Affiliations:** ^1^ School of Environmental Sciences University of Liverpool Liverpool UK; ^2^ School of Mathematical and Physical Sciences Sheffield UK; ^3^ Centre for Ecology and Conservation University of Exeter Cornwall UK; ^4^ Département Des Sciences Biologiques Université du Québec à Montréal Québec Canada; ^5^ British Antarctic Survey Natural Environment Research Council Cambridge UK; ^6^ Centre d'Etudes Biologiques de Chizé CNRS‐La Rochelle Université Villiers‐en‐Bois France; ^7^ Institute of Alpine and Arctic Research University of Colorado Boulder Boulder Colorado USA; ^8^ Biology Department Woods Hole Oceanographic Institution Woods Hole Massachusetts USA

**Keywords:** black‐browed albatross, environment, life‐history strategies, pace‐of‐life, reproductive senescence, *Thalassarche melanophris*

## Abstract

Individuals face a trade‐off between allocating resources to reproduction or self‐maintenance, yet the drivers of the existence and strength of such trade‐off have been hard to determine. Environmental conditions are thought to play a crucial role, as long‐lived species are predicted to favour more precautionary life‐history strategies in variable environments. However, empirical evidence remains limited. Using long‐term monitoring of two black‐browed albatross *Thalassarche melanophris* populations, we investigated variation in life‐history strategies under contrasting environmental conditions, through reproductive senescence. In more variable environments, individuals displayed generally slower life histories (i.e., slow, late‐onset senescence) and greater among‐individual variation in life‐history strategies. Interestingly, earlier and faster reproductive senescence correlated with higher lifetime reproductive success regardless of environmental variability, suggesting that either faster life histories incur higher fitness or successful reproduction accelerates reproductive senescence. These findings reveal how environmental variability shapes life‐history strategies, highlighting potential responses to increasing environmental variability in a changing world.

## Graphical abstract

Life‐history theory predicts a trade‐off between allocation of resources to reproduction or self‐maintenance, yet how environmental variability shapes this trade‐off remains poorly understood. We show that black‐browed albatrosses ( *Thalassarche melanophris* ) breeding in more variable environments display slower life histories (slow, late‐onset reproductive senescence) and greater among‐individual variation in life‐history strategies. However, earlier and faster reproductive senescence correlates with higher lifetime reproductive success regardless of environmental variability.

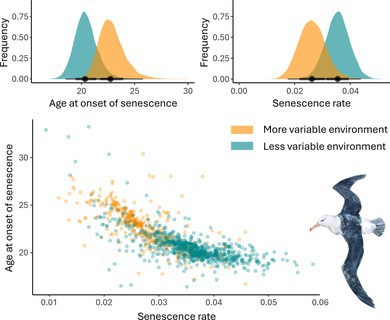

## 1 Introduction

Life‐history theory states that individuals face a trade‐off between allocating resources to reproduction and self‐maintenance (Stearns 1992; 1989; Williams 1966). Investment in current reproduction can result in fitness costs paid later in life (Lemaître et al. 2015; Reznick 1992), through a reduction in future reproductive performance, termed *reproductive senescence* or in survival, termed *actuarial senescence* (Kirkwood 1977; Kirkwood and Austad 2000). The plasticity shown by individuals in adjusting their investment in current reproduction to resolve life‐history trade‐offs, coupled with selection, often leads to covariation between traits that forms a fast‐slow continuum of life‐history strategies (D. Roff 1993; D. A. Roff 2002; Stearns 1992). At one end of this continuum are faster life histories, characterised by increased allocation to current reproduction. They are associated with a younger age at first reproduction, higher reproductive rates, lower survival rates (Stearns 1992; 1983), and a faster, earlier‐onset senescence (Jones et al. 2008; Péron et al. 2010; van De Walle et al. 2023). At the other end are slower life histories, where individuals prioritise survival over reproduction. This leads to an older age at first reproduction, lower reproductive rates, higher survival rates (Stearns 1992, 1983), and a slower, later‐onset senescence (Jones et al. 2008; Péron et al. 2010; van De Walle et al. 2023).

Environmental conditions exert selective pressures on life‐history strategies (Hämäläinen et al. 2021; Hastings and Caswell 1979; Roff 2001; Tuljapurkar et al. 2009). For instance, altitudinal, latitudinal or climatic gradients often shape variation in life histories among species and populations (Bastianelli et al. 2017; Boyle et al. 2016; Cayuela et al. 2021; Hille and Cooper 2015; Laiolo et al. 2015). The harshness and variability of the environment are expected to drive the evolution of life‐history strategies (Brumbach et al. 2009; Hämäläinen et al. 2021; Stearns 1992). Environmental conditions may select for contrasting life‐history strategies depending on the life stage on which they act (Ellis et al. 2009; Stearns 1976). Indeed, when stochastic or unfavourable environmental conditions reduce adult survival, faster life histories are predicted to evolve, through plasticity or selection (Ellis et al. 2009; Stearns 1976), that is, individuals increase allocation to current reproduction at a cost to their future reproduction and/or survival. This is mirrored in a faster, earlier‐onset senescence (Cayuela et al. 2020). In contrast, slower life histories are favoured when environmental harshness or stochasticity decreases offspring survival (Ellis et al. 2009; Stearns 1976). Individuals are predicted to reduce allocation to current reproduction to increase their survivorship and allocation to future reproduction (Wilbur and Rudolf 2006). Fitness can thus be maximised by spreading offspring mortality risk across a higher number of breeding attempts, that is, through bet‐hedging (Wilbur and Rudolf 2006). As reducing allocation to current reproduction induces a slower accumulation of reproductive costs over a longer lifespan, this should translate into delayed and slower senescence.

Many authors have suggested that environmental constraints may limit resources available to individuals, therefore strengthening life‐history trade‐offs within populations (Hämäläinen et al. 2021), whereas high‐quality environments may instead provide sufficient resources to mask life‐history trade‐offs (Lemaître et al. 2015; Nussey et al. 2013). Furthermore, within a population, individuals may respond differently to environmental constraints, leading to greater individual variation in life‐history strategies (Mathot and Frankenhuis 2018; van Noordwijk and de Jong 1986). Such variation has, for example, been linked to individual ability to acquire resources in the environment (van Noordwijk and de Jong 1986) or environmental conditions experienced during early life (Cooper and Kruuk 2018; Grafen 1988) and is further reflected in among‐individual variation in reproductive senescence (Bouwhuis et al. 2010; Nussey et al. 2007; Patrick and Weimerskirch 2015). Theory suggests that both environmental harshness and variability should lead to an increase in among‐individual variance in life‐history strategies (O'Dea et al. 2016; Roff 2002), but empirical evidence is scarce (Hämäläinen et al. 2021), especially regarding reproductive senescence (Lemaître et al. 2020; Valenzuela‐Sánchez et al. 2023). Indeed, while the onset and rate of reproductive senescence are predicted to be linked to the position of the species, population or individual along the fast‐slow continuum of life‐history strategies (Jones et al. 2008; Lemaître et al. 2020), the link between environmental conditions and reproductive senescence remains to be assessed, raising the need to study populations living in contrasting environments (Lemaître et al. 2020).

Long‐lived species are ideal models for investigating how environmental variability affects reproductive senescence and hence variation in life‐history strategies among and within populations. Because their high survival rates are buffered against environmental fluctuations (Gaillard and Yoccoz 2003; Sæther and Bakke 2000), these species are expected to cope with environmental variation by adjusting allocation to current or future reproduction (Hamel et al. 2010). This is reflected in a higher variance in allocation to reproduction in long‐lived than short‐lived species (Hamel et al. 2010), which may translate into variation in reproductive senescence. In this study, we analysed data from long‐term monitoring of marked individuals in two populations of black‐browed albatrosses *Thalassarche melanophris* breeding in the southern hemisphere under contrasting environmental conditions. At Bird Island (South Georgia), environmental and oceanographic conditions are highly variable because of the influence of large‐scale climatic processes such as the El Niño‐Southern Oscillation (ENSO) or Southern Annular Mode (SAM) (Murphy et al. 2007; Trathan et al. 2012, 2003). There, black‐browed albatross diet includes variable but generally high proportions of fish and Antarctic krill *Euphausia superba* (Mills et al. 2020), and the abundance of krill fluctuates greatly between years (Fielding et al. 2014). In contrast, at the Kerguelen archipelago, environmental and oceanographic conditions are less variable (Nevoux, Forcada, et al. 2010), ENSO influence is limited and climate variability is primarily driven by SAM (Pohl et al. 2021). At Kerguelen, black‐browed albatrosses rely on less spatially and temporally variable prey during the breeding season (fish, penguin carrion and cephalopods; Cherel et al. 2000). Previous research demonstrated that black‐browed albatrosses at Bird Island have higher survival rates and lower breeding success than at Kerguelen (Nevoux, Forcada, et al. 2010), suggesting that the former may rely on bet‐hedging strategies by reducing allocation to current reproduction and diluting offspring mortality risk across more breeding attempts. While previous studies have compared some aspects of life‐history strategies such as survival and breeding success at the population level, none has examined how environmental conditions shape other key life‐history traits such as individual variation in the age at onset and rate of reproductive senescence.

To shed light on the effect of environmental variability on intraspecific variation in life‐history strategies, we compared age‐specific changes in reproductive performance within and between these two populations of black‐browed albatrosses. (1) We tested whether black‐browed albatrosses breeding at Bird Island—where the environment is more variable—display slower life‐history strategy on average, mirrored in a later onset of reproductive senescence and slower senescence rates, compared to black‐browed albatrosses breeding at Kerguelen. (2) Within populations, we tested for individual variation in life‐history strategies along a fast‐slow continuum, by looking at the relationship between onset and rate of senescence. (3) We investigated whether environmental variability was associated with higher variation among individuals in life‐history strategies. (4) We evaluated the fitness outcomes of the life‐history strategies exhibited by individuals from the two populations. Specifically, we predicted (H_0_) that fast‐slow life‐history strategies would be associated with equal lifetime reproductive success (the number of chicks fledged by an individual over its lifetime, a proxy for individual fitness) within populations, that is, that alternative strategies have evolved.

## 2 Material and Methods

### 2.1 Monitoring of Black‐Browed Albatrosses

Black‐browed albatrosses are long‐lived and initiate breeding for the first time on average at ca 10 years old, with some variation among populations (Arnold et al. 2006; Nevoux, Weimerskirch, and Barbraud 2010; Weimerskirch et al. 1987). Black‐browed albatrosses produce a single egg and are annual breeders. The long‐term monitoring of black‐browed albatrosses has been carried out at Canyon des Sourcils Noirs (49°41′ S, 70°14′ E), Kerguelen archipelago, and at Bird Island (54°00′ S, 38°03′ E), South Georgia. Ringing of chicks started in austral summer 1967/68 (Kerguelen) and 1958/59 (Bird Island) with consistent annual monitoring since 1979/80 (Kerguelen) and 1975/76 (Bird Island) (see Nevoux et al. 2007 and Pardo et al. 2017 for further description of field protocols at Kerguelen and Bird Island, respectively). All breeding adults are ringed with metal (stainless‐steel or alloy) and plastic rings with unique alphanumeric codes, allowing identification of individuals in consecutive seasons. Chicks in study colonies are ringed with a metal ring and so many adults are of known age. Every breeding season, nests were marked, the identities of both members of each breeding pair were recorded, and checks carried out to assess reproductive success, that is, whether the pair succeeded or failed to rear a chick to fledging.

To obtain a similar range of longitudinal data in the two populations, we only kept in the analyses known‐age birds fledged since austral summer 1975/76. As most birds start breeding between 5 and 15 years of age at Kerguelen (Nevoux, Weimerskirch, and Barbraud 2010), or 7 and 17 years of age at Bird Island (Arnold et al. 2006; Prince et al. 1994), all individuals that bred for the first time before reaching a threshold of 20 years old were included in the analyses. This excluded 27 birds (likely ring‐reading errors or chronic nonbreeders). We considered individuals dead if not seen during the last 4 years of the study, given the high site fidelity of established breeders and because individuals rarely take longer than three years between breeding attempts (< 2.3% and 1.7% observations at Kerguelen and Bird Island, respectively). Our dataset included 5854 breeding attempts from 874 individuals at Kerguelen and 2648 breeding attempts from 360 individuals at Bird Island between 1975/76 and 2023/24. Of these, 467 and 161 individuals were presumed dead at the end of the monitoring period at Kerguelen and Bird Island, respectively. Given the overlap in body measurements of black‐browed albatrosses, sex cannot be recorded in the field, and we therefore did not account for sex effects in our analyses.

### 2.2 Statistical Analyses

Statistical analyses were conducted in R 4.3.1. (R Core Team 2023) using the brms package (Bürkner 2017). Models were run with eight chains of 4000 iterations including a 2000 iteration warm‐up. We used weakly informative priors in the models with: normal priors ($N\left[0,5\right]$ and $N\left[0,2\right]$, respectively) for intercepts and fixed effects, Student‐t priors (df = 3, mean = 0, scale = 1) for group‐level standard deviations and LKJ (Lewandowski–Kurowicka–Joe) priors (*η* = 2) for correlations among random intercepts and slopes, which weakly favour near‐zero correlations. Models were also tested with flat priors that provided similar results. All continuous variables were mean‐centred and scaled to standard deviation units. We verified that the model met all assumptions on convergence and autocorrelation. Posterior predictive checks were used to determine if the model fitted the observed data (Gelman et al. 2013). We report the posterior mean for each parameter and population comparison with the highest posterior density interval (HPDI) at 95%. To verify the robustness of our analyses against individuals with few breeding attempts, we conducted similar analyses on a subset of birds recorded breeding at least four times during the study period (n_Ker_ = 5341, n_ID Ker_ = 551, n_BI_ = 2439, n_ID BI_ = 247). These analyses gave qualitatively similar results, presented in Supporting Information S1.

#### 2.2.1 Variation in Life‐History Strategy Between Populations

To test for intraspecific variation in annual changes in reproductive performance with age, we built a generalised linear mixed model (GLMM) with a binomial error distribution with reproductive success as the dependent variable and population (Bird Island or Kerguelen), age (as a linear and quadratic effect) and the two‐way interactions between these two age components and population as independent variables. Because individuals breeding for the first time perform poorly compared with experienced ones (Nevoux et al. 2007; Ventura et al. 2021), we added a categorical variable to control for primiparity or multiparity. To account for the non‐independence of birds monitored during a breeding season and to quantify the effect of environmental variability on reproductive success in each population, we included year as a random intercept in the models. Cohort was also included as a random intercept to control for the non‐independence of birds fledged in the same year. To assess variation among individuals in life‐history strategy, we included a random intercept for individual identity and random slopes for the linear and quadratic age terms, hence allowing individuals to differ in ageing pattern. For each random effect, we used a separate covariance matrix for each population. The equation of the model is presented in Supporting Information S2.

#### 2.2.2 Among‐Individual Variation in Life‐History Strategy

**FIGURE 1:**
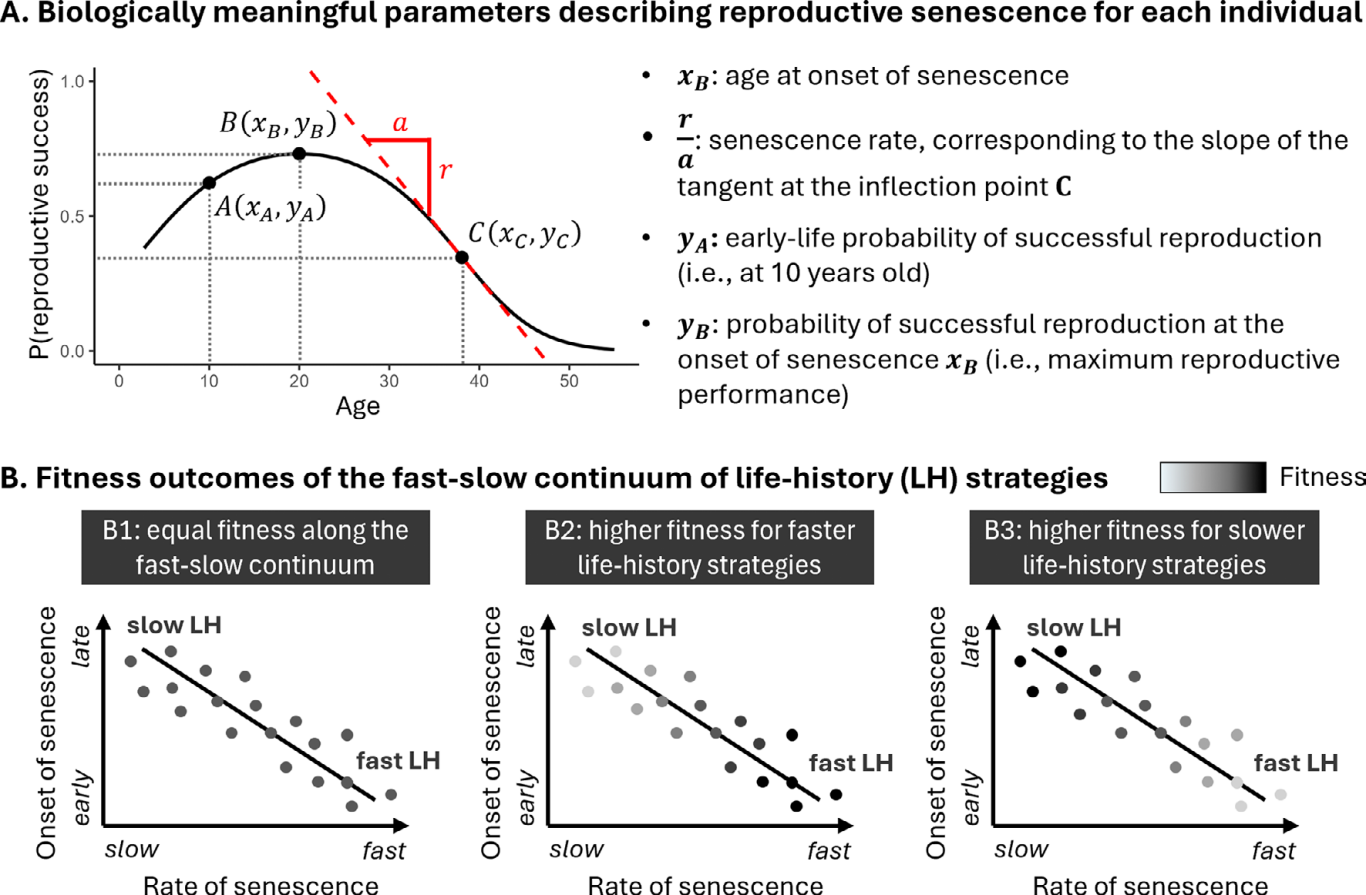
Methodological framework. (A) For each individual, we derived biologically meaningful parameters describing the pattern of reproductive senescence, based on the random slope and intercept generalised linear mixed model investigating variation in reproductive success with age (see 2.2.1). These parameters were age at the onset of senescence, senescence rate, early‐life probability of successful reproduction and probability of successful reproduction at the onset of senescence. (B) The relationship between the onset and rate of senescence characterises the position of the individual along the fast‐slow continuum of life‐history strategies. Equal fitness along this continuum is expected under the assumption of evolution of alternative strategies within populations (B1). Alternatively, faster (B2) or slower (B3) life‐history strategies could be associated with higher fitness.

To compare life‐history strategies within and among the two populations of black‐browed albatrosses, we extracted the estimated individual‐level values of intercept, linear and quadratic age effects, obtained from the GLMM in Section 2.2.1. Using these estimated values, we derived four biologically meaningful life‐history parameters for each individual: age at the onset of senescence, senescence rate, early‐life performance and performance at the onset of senescence (Figure 1A, Supporting Information S2). Estimated age at onset of senescence was defined as the age at the peak of the quadratic curve of reproductive success versus age. We defined estimated senescence rate as the slope of the tangent at the inflection point of this curve. The age at this inflection point is, by definition, the age at which the senescence was the fastest. We defined early‐life performance as the probability of successful reproduction at 10 years old (corresponding to the average age at first reproduction in the two populations: Bird Island: 10.2 ± 2.2 SD; Kerguelen: 9.9 ± 2.5 SD). Finally, we defined performance at the onset of senescence as the probability of successful reproduction at the age at onset of senescence (i.e., at the peak of the curve), and used it as a proxy for maximum individual performance.

Then, we compared the posterior distributions of these four life‐history parameters between the two populations using the function ‘boot.overlap’ (overlapping package, Pastore 2018). We also measured the variance of each parameter for each population to assess whether environmental variability is associated with higher among‐individual variance in life‐history strategies within populations. Finally, we tested for variation among individuals in life‐history strategies along a fast‐slow continuum by looking at the correlation between individual estimated age at onset of senescence and estimated senescence rate in each population. One individual characterised by little to no senescence (estimated onset of senescence after 40 year old; see Supporting Information S3 for further details) was removed from the analyses.

#### 2.2.3 Fitness Outcomes of Intraspecific Variation in Life‐History Strategy

We compared the fitness outcomes of the life‐history strategies exhibited by black‐browed albatrosses in the two populations (Figure 1B). For this, we analysed the subset of individuals fledged before 2000 and presumed dead by the end of the study period (n_ID Ker_ = 380, n_ID BI_ = 125) and investigated the association between lifetime reproductive success (i.e., the total number of chicks fledged by a given individual over its lifetime) and the onset and rate of senescence. Additionally, we verified that the relationships we observed when investigating the association between fitness and life‐history strategies were linked to the costs of successfully breeding rather than the cost of attempting breeding by running the same analysis and replacing lifetime reproductive success by the number of breeding attempts in the lifetime (Supporting Information S4). To ensure that the analyses were not biased towards birds with a short lifespan—either through stochastic events or incidental mortality (bycatch) in fisheries (Michael et al. 2017) rather than ageing—the following analyses were also carried out with the entire dataset (n_ID Ker_ = 874, n_ID BI_ = 360), distinguishing between birds that were presumed dead versus those still alive, as lifetime reproductive success is likely underestimated for the latter. These analyses gave qualitatively similar results, detailed in Supporting Information S5.

We first built a generalised linear model (GLM) with a Poisson error distribution with individual lifetime reproductive success as the dependent variable and estimated age at the onset of senescence, population and the two‐way interaction between population and estimated age at the onset of senescence as independent variables. We then built a GLM with a Poisson error distribution with individual lifetime reproductive success as the dependent variable and estimated senescence rate, population and the two‐way interaction between population and estimated senescence rate as independent variables. Estimated age at the onset of senescence and senescence rate were not included in the same model because of the high correlation between the two variables (correlations, r_BI_ = −0.65; r_Ker_ = −0.72). These models allowed us to test the association between individual fitness and the onset and rate of reproductive senescence, while allowing the relationship to differ between populations. The robustness of the results was confirmed by propagating error uncertainty in the estimation of age at onset and rate of senescence. To do so, we re‐ran each GLM 2000 times, using 2000 posterior estimates of age at the onset of senescence and senescence rate, randomly selected from the 95% credibility intervals of their posterior distribution (Supporting Information S6).

## 3 Results

### 3.1 Variation in Life‐History Strategy Between Populations and Among Individuals

**FIGURE 2:**
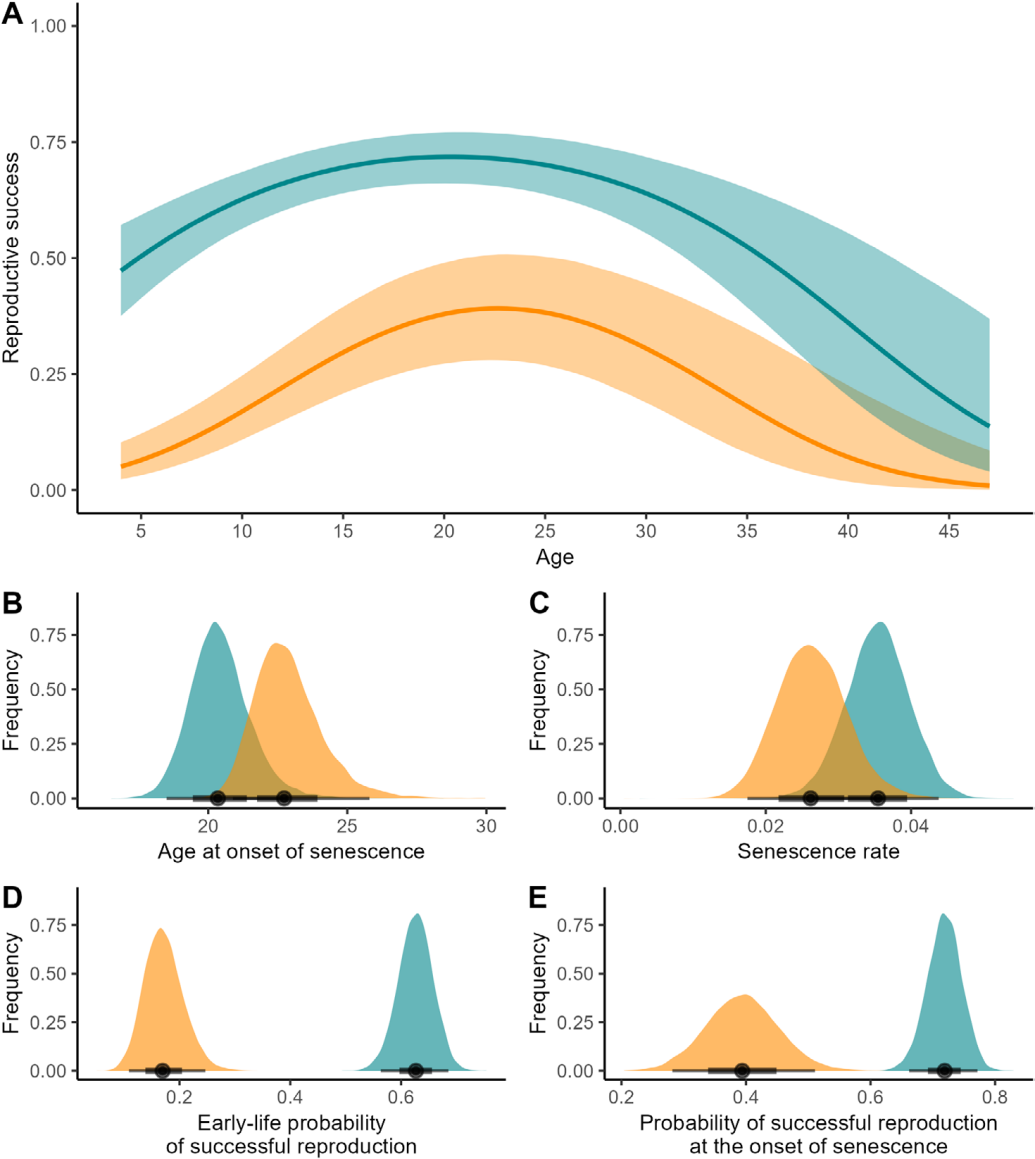
(A) Relationship between average population‐level reproductive success and age and posterior distribution for each population of estimated (B) age at the onset of senescence, (C) senescence rate, (D) early‐life probability of successful reproduction (i.e., at 10 years old) and (E) probability of successful reproduction at the onset of senescence in black‐browed albatrosses breeding at Bird Island (in orange) and Kerguelen (in blue). In (A) lines depict population‐level predictions and shaded areas account for 95% confidence interval. In (B–E) areas depict posterior probability distributions.

Reproductive success increased with age before decreasing at old age in both populations. However, black‐browed albatrosses breeding at Kerguelen and Bird Island differed in the shape of their reproductive ageing pattern (Table 1, Figure 2A). Individuals from Kerguelen—where the environment is less variable—displayed higher breeding success (intercept estimate: 0.87 (0.63, 1.14)) than individuals from Bird Island (intercept estimate: −0.73 (−1.19, −0.29)). Individuals from Kerguelen also displayed a lower linear age slope (0.20 (0.10, 0.30)) and a higher quadratic age slope (−0.15 (−0.21, −0.09)) than those from Bird Island (linear age effect: 0.56 (0.39, 0.74), quadratic age effect: −0.27 (−0.38, −0.16)). Primiparous breeders displayed a lower probability of successful reproduction (Table 1). In terms of key biological parameters, estimated age at the onset of senescence was higher at Bird Island than at Kerguelen, with an overlap of 25.53% ± 0.35% of posterior distributions (Figure 2B). Estimated senescence rates were lower at Bird Island than at Kerguelen, with an overlap of 32.34% ± 0.42% of posterior distributions (Figure 2C). Estimated early‐life and maximum reproductive performances were both higher at Kerguelen than at Bird Island, with little to no overlap between the posterior distributions of the two life‐history parameters (estimated early‐life probability of successful reproduction: overlap ± SE = 0.00% ± 0.00%, Figure 2D; estimated probability of successful reproduction at the onset of senescence: overlap ± SE = 0.04% ± 0.01%, Figure 2E).

**TABLE 1:** Parameter estimates (β) and 95% credible intervals (CI) from the random slope and intercept generalised linear mixed model with a binomial error distribution explaining variation in black‐browed albatross reproductive success in response to age (as a linear and quadratic effect), population (Bird Island or Kerguelen) and primiparity or multiparity of the breeding attempt. Individual identity, year and cohort were included as random intercepts linear and quadratic age terms were included as random slopes for each individual.

	Estimate (β)	95% credible intervals (CI)
Fixed effects		
Intercept	−0.73	(−1.19, −0.29)
Age	0.56	(0.39, 0.74)
Age^2^	−0.27	(−0.38, −0.16)
Population: Kerguelen	1.60	(1.11, 2.20)
Age × population: Kerguelen	−0.37	(−0.57, −0.18)
Age^2^ × population: Kerguelen	0.12	(0.00, 0.24)
Breeding attempt: primiparity	−0.48	(−0.65, −0.30)
Random effects		
SD of random intercepts on ID: Bird Island	0.72	(0.53, 0.92)
SD of random intercepts on ID: Kerguelen	0.58	(0.44, 0.72)
SD of random slopes on age per ID: Bird Island	0.37	(0.07, 0.62)
SD of random slopes on age per ID: Kerguelen	0.19	(0.01, 0.39)
SD of random slopes on age^2^ per ID: Bird Island	0.12	(0.01, 0.29)
SD of random slopes on age^2^ per ID: Kerguelen	0.13	(0.01, 0.26)
SD of random intercepts on year: Bird Island	1.21	(0.90, 1.63)
SD of random intercepts on year: Kerguelen	0.70	(0.54, 0.92)
SD of random intercepts on cohort: Bird Island	0.13	(0.01, 0.34)
SD of random intercepts on cohort: Kerguelen	0.10	(0.01, 0.24)
Correlations between random slopes and intercepts		
Correlation between random intercept and random age slopes: Bird Island	−0.32	(−0.79, 0.28)
Correlation between random intercept and random age slopes: Kerguelen	0.04	(−0.59, 0.64)
Correlation between random intercept and random age^2^ slopes: Bird Island	−0.40	(−0.90, 0.47)
Correlation between random intercept and random age^2^ slopes: Kerguelen	−0.47	(−0.86, 0.30)
Correlation between random age and age^2^ slopes: Bird Island	−0.10	(−0.76, 0.70)
Correlation between random age and age^2^ slopes: Kerguelen	−0.23	(−0.84, 0.63)

**FIGURE 3:**
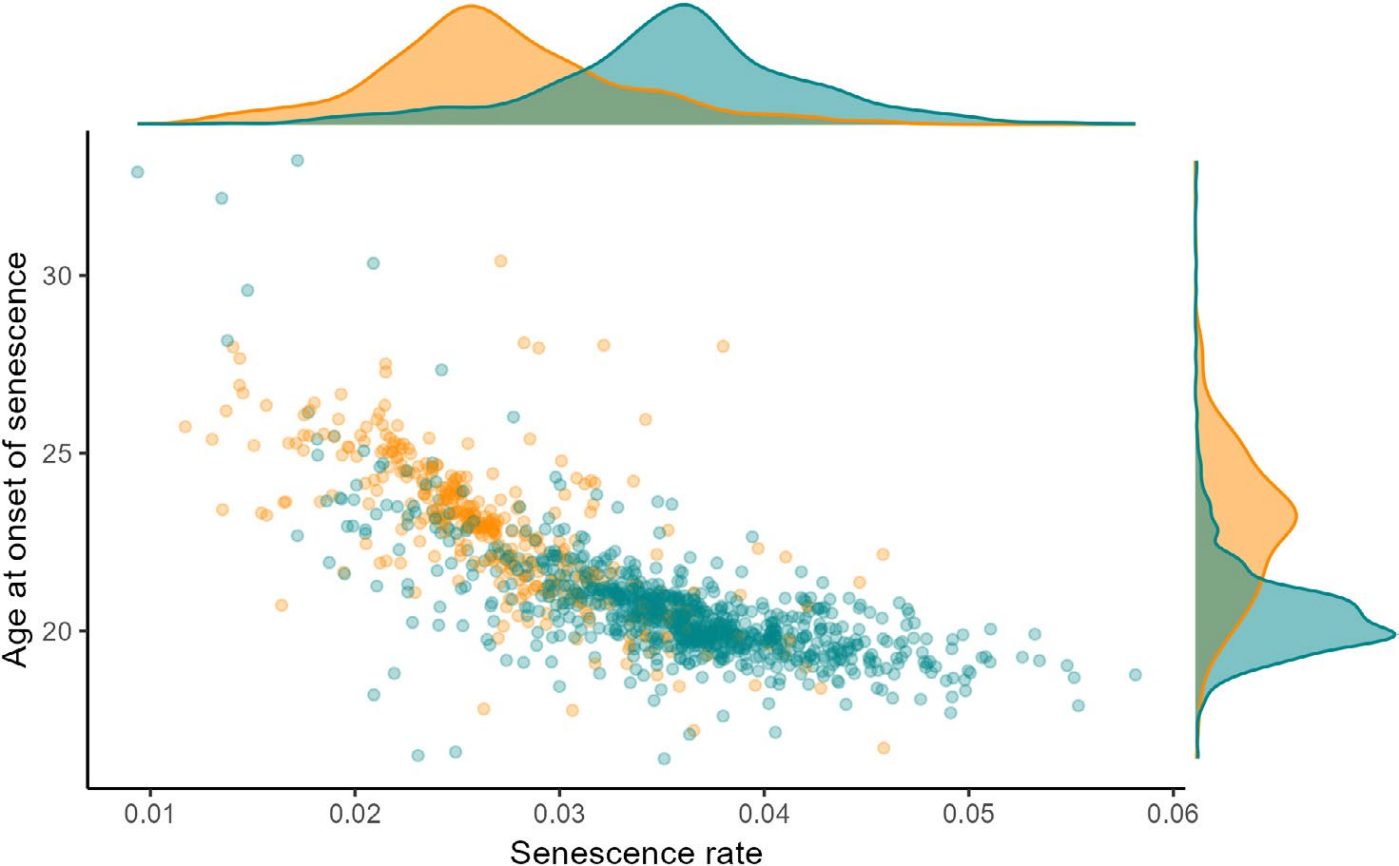
Relationship between individual estimated age at the onset of senescence and estimated senescence rate in black‐browed albatrosses breeding at Bird Island (in orange) and Kerguelen (in blue). Also shown are the density plots of these two variables for each population.

Most life‐history trait variances were higher at Bird Island than at Kerguelen, the ratio of variance at Bird Island over variance at Kerguelen ranging from 1.22 (estimated senescence rate) to 4.45 (estimated probability of successful reproduction at the onset of senescence; details in Table 2). The among‐year variance in reproductive success was 2.99 times higher at Bird Island than at Kerguelen (Table 2), and the among‐cohort variance in reproductive success was 1.77 times higher at Bird Island than at Kerguelen (Table 2). Individual estimated ages at the onset of senescence were negatively associated with estimated senescence rates, both at Bird Island and Kerguelen (correlations, r_BI_ = −0.70; r_Ker_ = −0.70, Figure 3, Supporting Information S3).

**TABLE 2:** Among‐year and among‐cohort variance in reproductive success and among‐individual variance in life‐history traits (predicted age at onset of senescence, senescence rate, early‐life probability of successful reproduction and probability of successful reproduction at the onset of senescence) at Bird Island and Kerguelen, and variance ratio between the two colonies.

Parameter	Bird Island	Kerguelen	Ratio
Among‐year variance in reproductive success (random intercept on year)	1.509	0.504	2.993
Among‐cohort variance in reproductive success (random intercept on cohort)	0.026	0.015	1.767
Predicted age at the onset of senescence	0.047	0.032	1.460
Predicted senescence rate	0.0009	0.0007	1.225
Predicted early‐life probability of successful reproduction	0.0012	0.0009	1.301
Predicted probability of successful reproduction at the onset of senescence	0.0033	0.0008	4.445

### 3.2 Fitness Outcomes of Intraspecific Variation in Life‐History Strategy

**FIGURE 4:**
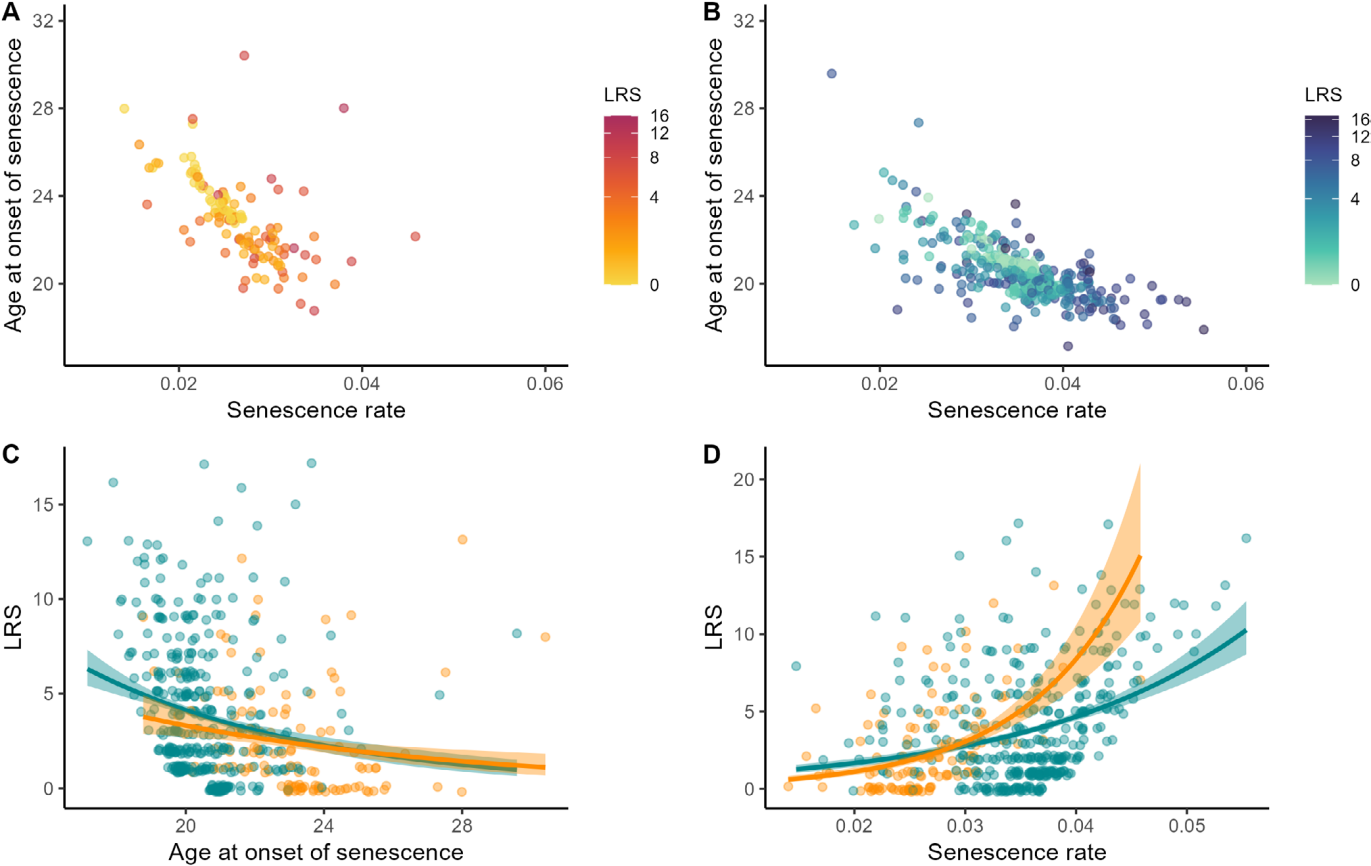
Relationship between individual estimated age at the onset of senescence, estimated senescence rate and lifetime reproductive success (colour gradient) in black‐browed albatrosses breeding at (A) Bird Island and (B) Kerguelen fledged before 2000 and presumed dead by the end of the study period. Relationship between lifetime reproductive success and (C) estimated age at the onset of senescence, (D) estimated senescence rate in black‐browed albatrosses breeding at Bird Island (in orange) and Kerguelen (in blue) fledged before 2000 and presumed dead by the end of the study period. Lines depict population‐level responses and shaded areas account for 95% confidence interval.

Individual lifetime reproductive success was negatively associated with estimated age at the onset of senescence at Bird Island (estimate: −0.20 (−0.31, −0.08), Figures 4A,C) and Kerguelen (estimate: −0.28 (−0.36, −0.20), Figure 4B,C), with no clear difference in the strength of the relationship between the two populations (interaction term estimate: −0.08 (−0.23, 0.06)). This result indicates that an earlier onset of senescence correlates with higher fitness. Lifetime reproductive success was positively associated with estimated senescence rate both at Bird Island (estimate: 0.69 (0.56, 0.82), Figure 4A,D) and Kerguelen (estimate: 0.36 (0.30, 0.42), Figure 4B,D), with a stronger relationship at Bird Island than at Kerguelen (interaction term estimate: −0.33 (−0.47, −0.19)). This result suggests stronger senescence rates in individuals producing more offspring during their lifetime.

## 4 Discussion

Using extensive long‐term monitoring data (1975–2024) from two populations of black‐browed albatrosses breeding under contrasting environmental conditions, we provide evidence for intraspecific variation in life‐history strategies, with individuals breeding under more variable environmental conditions (i.e., at Bird Island) displaying slower life‐history strategies—reflected in a later onset and slower rate of reproductive senescence. Previous studies have reported reproductive senescence in both study populations (Froy et al. 2017; Pardo et al. 2013) and suggested that the differences in life‐history strategies were driven by the contrasting environments (Nevoux, Forcada, et al. 2010). However, the extent to which environmental conditions shape reproductive senescence among populations and individuals had been overlooked (Lemaître et al. 2020). Additionally, we found higher variance in life‐history traits among individuals at Bird Island, aligning with the theory that environmental harshness or variability should lead to increased among‐individual variance in life‐history strategies (Hämäläinen et al. 2021; O'Dea et al. 2016; Roff 2002). Contrary to the prediction that a fast‐slow life‐history continuum would be maintained within populations through equal fitness of alternative strategies, we showed that within both populations, individuals displaying a faster pace‐of‐life—through earlier and stronger reproductive senescence—had the highest fitness. This finding could be attributed to a higher ability of individuals displaying faster life‐history strategies to acquire resources or to reproductive senescence arising from accumulated costs of reproduction.

### 4.1 Variation in Reproductive Senescence Between Populations

We found that annual variation in reproductive success was three times higher at the colony characterised by higher environmental variability (Bird Island) than at the one with less environmental variability (Kerguelen). The magnitude of interannual variation in breeding success is in accordance with a previous study which showed that sea surface temperatures were three times more variable at Bird Island than Kerguelen (Nevoux, Forcada, et al. 2010). In addition, fluctuating levels of egg or chick predation also contribute to the variability in breeding outcomes for black‐browed albatrosses breeding at Bird Island (Catry et al. 2010; Forster and Phillips 2009). Egg predation in particular will be higher in years of poor food availability because albatrosses that start their incubation stint in poor body condition or whose partner takes a long time to return are more likely to desert the egg (Forster and Phillips 2009; Pinaud and Weimerskirch 2002).

We showed that the patterns of reproductive senescence differed between the two study populations. Black‐browed albatrosses breeding at Bird Island—where the environment is more variable—displayed a slower, later‐onset reproductive senescence than those breeding at Kerguelen. Considering previous research linking the onset and rate of reproductive senescence to the position of the species, population or individual along the fast‐slow continuum of life‐history strategies (Jones et al. 2008; Lemaître et al. 2020), this result indicates that breeding under more variable environmental conditions was associated with slower life‐history strategies. While intraspecific variation in patterns of actuarial senescence has been highlighted in populations differing in environmental conditions (e.g., Bleu et al. 2015; Bryant and Reznick 2004; Cayuela et al. 2021, 2020; Tully et al. 2020; Valenzuela‐Sánchez et al. 2023) only a few studies have, to our knowledge, tested for intraspecific variation in reproductive senescence in wild populations (e.g., Austad 1993; Benoît et al. 2018; Bérubé et al. 1999; Blažek et al. 2017; Le Coeur et al. 2024; Neves et al. 2024; Sparkman et al. 2007; Tully et al. 2020; Vrtílek et al. 2018), and rarely under contrasting environmental conditions. This study is among the first to reveal population‐level variation in patterns of reproductive senescence across different levels of variability of environmental conditions.

Coupled with higher survival (Nevoux, Forcada, et al. 2010), the lower breeding performance—both during early life and at the onset of senescence—of black‐browed albatrosses breeding at Bird Island, supports the hypothesis that they prioritise investment in survival over reproduction more than birds at Kerguelen. Such prioritisation should lead to a slower accumulation of reproductive costs over the lifetime, likely reflected in the observed later onset of reproductive senescence and slower senescence rates. Further support for this hypothesis is the steeper slope of the relationship between lifetime reproductive success and senescence rate at Bird Island than at Kerguelen (Figure 4D), which suggests a lower cost of offspring production. When variable or unfavourable environmental conditions reduce offspring survival, as at Bird Island, individuals may therefore maximise fitness through bet‐hedging (Wilbur and Rudolf 2006), that is, by reducing reproductive investment and spreading offspring mortality risk across a higher number of breeding attempts.

### 4.2 Among‐Individual Variation in Reproductive Senescence and Fitness

We found higher among‐individual variance in all parameters of interest (age at onset of senescence, senescence rate, early‐life and maximum reproductive performance) in black‐browed albatrosses breeding at Bird Island, under more variable environmental conditions, than at Kerguelen. This finding is in line with the hypothesis that unfavourable or variable environmental conditions might increase among‐individual variance in life‐history strategies (Hämäläinen et al. 2021; O'Dea et al. 2016; Roff 2002). In both populations, the negative correlation between estimated age at onset of senescence and senescence rate suggested among‐individual variation along the fast‐slow continuum of life‐history strategies, ranging from faster life histories characterised by earlier and stronger reproductive senescence to slower life histories, characterised by later and slower reproductive senescence.

Given that natural selection should favour life‐history strategies that maximise fitness according to prevailing environmental conditions (Stott et al. 2024), we predicted that the existence of a fast‐slow continuum of life‐history strategies within populations would be the result of the evolution of alternative reproductive strategies yielding equal lifetime fitness. Contrary to our prediction, faster life‐histories—that is, earlier onset of senescence and stronger senescence rates—were associated with higher lifetime reproductive success in both populations. Higher fitness of individuals with fast life‐histories may seem counterintuitive in long‐lived species that have evolved slow life histories on average.

Two non‐mutually exclusive hypotheses could explain this result. First, faster life histories may correlate with other phenotypic traits yielding higher fitness. Indeed, in both populations, earlier onset of senescence and faster senescence rate correlated with higher estimated probability of reproducing successfully in early life and maximum performance (see correlation matrix in Supporting Information S7). Faster life‐histories may be associated with individuals having a higher ability to acquire resources in the environment (Haave‐Audet et al. 2022; Laskowski et al. 2021), thereby reducing reproductive costs or enhancing reproductive success. Life‐history, behavioural and physiological traits often covary, a concept formalised as the pace‐of‐life syndrome (Dammhahn et al. 2018; Réale et al. 2010). Individuals displaying a faster pace‐of‐life are predicted to be more risk‐prone—that is, bolder and more exploratory—than those with a slower pace‐of‐life (Dammhahn et al. 2018; Réale et al. 2010), and there is growing empirical evidence of a link between boldness and foraging strategies (Pereira et al. 2024; Traisnel and Pichegru 2019)—including in albatrosses (Gillies et al. 2023; Patrick et al. 2017; Patrick and Weimerskirch 2014). For instance, bolder individuals tend to invest more in explorative than exploitative behaviour (Gillies et al. 2023; Patrick et al. 2017) and have stronger competitive abilities (Patrick and Weimerskirch 2014; Webster et al. 2009). Higher offspring production in bolder individuals has also been reported (Collins et al. 2019; Eccard et al. 2022; Patrick et al. 2017; Smith and Blumstein 2008), although this relationship may be sex‐specific and context‐dependent (Clermont et al. 2023; Eccard et al. 2022; Patrick and Weimerskirch 2014; Smith and Blumstein 2008), therefore remaining debated. A greater ability to acquire resources could therefore mediate the observed positive relationship between faster life‐history strategies and fitness.

Second, variation in the onset and rate of reproductive senescence could mirror the outcome of accumulated costs of reproduction, rather than reflect alternative life‐history strategies. The disposable soma theory states that individuals face a trade‐off between investing in reproduction or self‐maintenance, and that allocating resources to reproduction will incur a cost later in life (Kirkwood 1977). Producing offspring is costly and individuals successfully rearing a higher number of chicks to fledging during their lifetime, but not necessarily engaging in a higher number of breeding attempts (Supporting Information S4), are likely to accumulate reproductive costs and pay them through earlier and faster senescence, as highlighted by this study. Accordingly, high reproductive investment in early life has been shown to translate into greater costs in late life, that is, faster reproductive or actuarial senescence (Bouwhuis et al. 2010; Kirkwood and Rose 1991; Nussey et al. 2006; Orell and Belda 2002; Reed et al. 2008; Williams et al. 2006). The correlation between earlier onset of senescence, faster senescence rate and both higher early‐life and peak breeding performance (Supporting Information S7) also supports this hypothesis. Our findings align with those of a previous study carried out on Atlantic puffins *Fratercula arctica* , another long‐lived seabird species, which reported lower rates of actuarial senescence in populations characterised by lower productivity (Landsem et al. 2023). Additionally, an experimental study provided evidence for a steeper decline in reproductive effort in old female cavy *Cavia aperea* engaging in continuous reproduction than in those with interrupted reproduction (Trillmich et al. 2019), highlighting an impact of reproductive costs on reproductive senescence.

## 5 Conclusion

By providing evidence for intraspecific variation in the pace and variance of life‐history strategies in contrasting environments, this study contributes to our understanding of how variability of environmental conditions may shape life histories. We only considered two black‐browed albatross populations, because datasets of comparable longevity do not exist for other colonies. Expanding to cover the range of environmental conditions experienced by the species would allow us to further decouple colony‐specific differences from patterns driven by harsh or variable environmental conditions. This is particularly relevant given the difference in population trends of the two study colonies, with a steep decline at Bird Island (Mackley et al. 2025), contrasting with the relatively stable trend at Kerguelen (Weimerskirch et al. 2018). Importantly, we here focused on allocation of resources to reproduction through reproductive success. Earlier and stronger reproductive senescence—reflecting faster life‐history strategies—correlated with higher lifetime reproductive output in both populations. While this result suggests higher fitness of individuals displaying faster life‐history strategies, future research should aim to measure resource acquisition and allocation to disentangle the extent to which among‐individual variation in reproductive senescence patterns is driven by differences in resource acquisition or accumulated costs of reproduction.

## Author Contributions

Conceptualisation: Bertille Mohring, Jonathan R. Potts, Alastair J. Wilson, Denis Réale, Richard A. Phillips, Henri Weimerskirch, Samantha C. Patrick. Funding acquisition: Jonathan R. Potts, Alastair J. Wilson, Denis Réale, Richard A. Phillips, Henri Weimerskirch, Samantha C. Patrick. Methodological design: Bertille Mohring, Jonathan R. Potts, Alastair J. Wilson, Denis Réale, Samantha C. Patrick. Data curation and management: Bertille Mohring, Richard A. Phillips, Henri Weimerskirch, Christophe Barbraud, Karine Delord, Ashley Bennison, Andrew G. Wood, Samuel Peroteau, Francesco Ventura, Etienne Rouby. Project administration: Samantha C. Patrick. Resources and data: Richard A. Phillips, Henri Weimerskirch, Christophe Barbraud. Formal analysis: Bertille Mohring, Jonathan R. Potts. Writing – original draft: Bertille Mohring, Jonathan R. Potts, Alastair J. Wilson, Denis Réale, Samantha C. Patrick. Writing – review and editing: Bertille Mohring, Jonathan R. Potts, Alastair J. Wilson, Denis Réale, Richard A. Phillips, Henri Weimerskirch, Christophe Barbraud, Karine Delord, Ashley Bennison, Andrew G. Wood, Samuel Peroteau, Francesco Ventura, Etienne Rouby, Samantha C. Patrick.

## Funding

This work was supported by Zone Atelier Antarctique. British Antarctic Survey. Institut Polaire Français Paul Emile Victor, Project Ornitho2e 109. Natural Environment Research Council, NE/X000680/1. Terres Australes et Antarctiques Françaises.

## Conflicts of Interest

The authors declare no conflicts of interest.

## Supporting information


**Figure S1:1.** (A) Relationship between average population‐level reproductive success and age and posterior distribution for each population of predicted (B) age at the onset of senescence, (C) senescence rate, (D) early‐life probability of successful reproduction (i.e., at 10 years old) and (E) probability of successful reproduction at the onset of senescence in a subset of black‐browed albatrosses breeding at Bird Island (in orange) and Kerguelen (in blue) and with at least four breeding attempts during the study period. In (A) lines depict population‐level predictions and shaded areas account for 95% confidence interval. In (B–E) areas depict posterior probability distributions.
**Table S1:1:** Parameter estimates (*β*) and 95% credible intervals (CI) from the random slope and intercept generalised linear mixed model with a binomial error distribution explaining variation in black‐browed albatross reproductive success in response to age (as a linear and quadratic effect), population (Bird Island or Kerguelen) and primiparity or multiparity of the breeding attempt. Individual identity, year and cohort were included as random intercepts linear and quadratic age terms were included as random slopes for each individual. The model was run on a subset of black‐browed albatrosses breeding at Bird Island and Kerguelen with at least four breeding attempts during the study period.
**Table S1:2:** Among‐year and among‐cohort variance in reproductive success and among‐individual variance in life‐history traits (predicted age at onset of senescence, senescence rate, early‐life probability of successful reproduction and probability of successful reproduction at the onset of senescence) at Bird Island and Kerguelen and variance ratio between the two colonies. The model was run on a subset of black‐browed albatrosses breeding at Bird Island and Kerguelen with at least four breeding attempts during the study period.
**Figure S1:2:** Relationship between individual predicted age at the onset of senescence and senescence rate in black‐browed albatrosses breeding at Bird Island (in orange) and Kerguelen (in blue) on a subset of individuals with at least four breeding attempts during the study period.
**Figure S1:3:** Relationship between individual predicted age at the onset of senescence, predicted senescence rate and lifetime reproductive success (colour gradient) in black‐browed albatrosses breeding at (A) Bird Island and (B) Kerguelen fledged before 2000 and presumed dead by the end of the study period. Relationship between lifetime reproductive success and (C) predicted age at the onset of senescence, (D) predicted senescence rate in black‐browed albatrosses breeding at Bird Island (in orange) and Kerguelen (in blue) fledged before 2000, and with at least four breeding attempts and presumed dead by the end of the study period (n_ID Ker_ = 219, n_ID BI_ = 80). Lines depict population‐level responses and shaded areas account for 95% confidence interval.
**Table S1:3:** Estimate (95% CI) of the effects of predicted onset of senescence and senescence rate lifetime reproductive success (for birds with at least four breeding attempts during the study period, presumed dead or still alive at the end of the study period). Estimates with 95% CI not overlapping 0 are shown in bold.
**Figure S1:4:** Relationship between lifetime reproductive success and (A) predicted age at the onset of senescence, (B) predicted senescence rate in black‐browed albatrosses breeding at Bird Island (in orange) and Kerguelen (in blue) on a subset of individuals with at least four breeding attempts during the study period, from all cohorts, either presumed dead (darker shade) or alive (lighter shade) by the end of the study period. Lines depict population‐level responses and shaded areas account for 95% confidence interval.
**Figure S2:1:** Curve depicting individual‐level variation in the probability of reproductive success with age. Point A is located on the curve at an age of 10 (corresponding to early life in albatrosses). Point B corresponds to the peak of the curve, marking the onset of senescence. Point C represents the first inflection point of the curve, where the rate of change in reproductive success is at its maximum. The dashed red line is the tangent at the inflection point and r/a is the senescence rate (i.e., the gradient of the tangent).
**Figure S3:1:** Relationship between individual predicted age at the onset of senescence and predicted senescence rate in black‐browed albatrosses breeding at Bird Island (in orange) and Kerguelen (in blue) when including the individual with no apparent reproductive senescence. Also shown are the density plots of these two variables for each population.
**Figure S4:1:** Relationship between the number of breeding attempts during an individual's lifetime and (A) predicted age at the onset of senescence, (B) predicted senescence rate in black‐browed albatrosses breeding at Bird Island (in orange) and Kerguelen (in blue) fledged before 2000 and presumed dead by the end of the study period. Lines depict population‐level responses and shaded areas account for 95% confidence interval.
**Table S4:1:** Estimate (95% CI) of the effects of predicted onset of senescence and senescence rate on the number of breeding attempts during an individual's lifetime (for birds presumed dead or still alive at the end of the study period). Estimates with 95% CI not overlapping 0 are shown in bold.
**Figure S4:2:** Relationship between the number of breeding attempts during an individual's lifetime and (A) predicted age at the onset of senescence, (B) predicted senescence rate in black‐browed albatrosses breeding at Bird Island (in orange) and Kerguelen (in blue) from all cohorts, either presumed dead (darker shade) or alive (lighter shade) by the end of the study period. Lines depict population‐level responses and shaded areas account for 95% confidence interval.
**Table S5:1:** Estimate (95% CI) of the effects of predicted onset of senescence and senescence rate on lifetime reproductive success for birds presumed dead or still alive at the end of the study period. Estimates with 95% CI not overlapping 0 are shown in bold.
**Figure S5:1:** Relationship between lifetime reproductive success (LRS) and (A) predicted age at the onset of senescence, (B) predicted senescence rate in black‐browed albatrosses breeding at Bird Island (in orange) and Kerguelen (in blue) from all cohorts, either presumed dead (darker shade) or alive (lighter shade) by the end of the study period. Lines depict population‐level responses and shaded areas account for 95% confidence interval.
**Figure S6:1:** Robustness analysis of the association between lifetime reproductive success and predicted age at onset of senescence (‘onset’) and senescence rate (‘rate’) for black‐browed albatrosses breeding at Bird Island (orange) and Kerguelen (blue). Points represent mean parameter estimate, with error bars showing the standard deviation (thick lines) and 95% credible interval (thin lines). The dashed vertical red line indicates zero.
**Figure S7:1:** Correlation between life‐history parameters for black‐browed albatrosses breeding at (A) Bird Island and (B) Kerguelen fledged before 2000 and presumed dead by the end of the study period.

## Data Availability

The data supporting this study are available at https://doi.org/10.5285/e2d734d6‐e632‐427e‐b302‐a206f92ebd63 for Bird Island (Phillips et al. 2026) and at https://doi.org/10.48579/PRO/DHHAP0 for Kerguelen (Mohring 2026). Code supporting the analyses and results can be accessed on Zenodo at: https://doi.org/10.5281/zenodo.17314406 (Mohring et al. 2026).
