# Supplementary material for: Environmental Variability Shapes Life‐History Trade‐Offs Within and Between Populations of a Long‐Lived Seabird
Source: Ecol Lett. 2026 Apr 16;29(4):e70384. doi: 10.1111/ele.70384 (PMC13087207; doi:10.1111/ele.70384)
Supplement: Supplementary file 1 — Figure S1:1. (A) Relationship between average population‐level reproductive success and age and posterior distribution for each population of predicted (B) age at the onset of senescence, (C) senescence rate, (D) early‐life probability of successful reproduction (i.e., at 10 years old) and (E) probability of successful reproduction at the onset of senescence in a subset of black‐browed albatrosses breeding at Bird Island (in orange) and Kerguelen (in blue) and with at least four breeding attempts during the study period. In (A) lines depict population‐level predictions and shaded areas account for 95% confidence interval. In (B–E) areas depict posterior probability distributions. Table S1:1: Parameter estimates (β) and 95% credible intervals (CI) from the random slope and intercept generalised linear mixed model with a binomial error distribution explaining variation in black‐browed albatross reproductive success in response to age (as a linear and quadratic effect), population (Bird Island or Kerguelen) and primiparity or multiparity of the breeding attempt. Individual identity, year and cohort were included as random intercepts linear and quadratic age terms were included as random slopes for each individual. The model was run on a subset of black‐browed albatrosses breeding at Bird Island and Kerguelen with at least four breeding attempts during the study period. Table S1:2: Among‐year and among‐cohort variance in reproductive success and among‐individual variance in life‐history traits (predicted age at onset of senescence, senescence rate, early‐life probability of successful reproduction and probability of successful reproduction at the onset of senescence) at Bird Island and Kerguelen and variance ratio between the two colonies. The model was run on a subset of black‐browed albatrosses breeding at Bird Island and Kerguelen with at least four breeding attempts during the study period. Figure S1:2: Relationship between individual predicted age at the onset of s [file ELE-29-0-s001.docx]

Supporting information for the manuscript:

Environmental variability shapes life-history trade-offs within and between populations of a long-lived seabird

Bertille Mohring^1^, Jonathan R. Potts^2^, Alastair J. Wilson^3^, Denis Réale^4^, Richard A. Phillips^5^, Henri Weimerskirch^6^, Christophe Barbraud^6^, Ashley Bennison^5^, Karine Delord^6^, Andrew G. Wood^5^, Samuel Peroteau^6^, Etienne Rouby^7^, Francesco Ventura^8^, Samantha C. Patrick^1^

^1^School of Environmental Sciences, University of Liverpool, Liverpool, UK

^2^School of Mathematical and Physical Sciences, Hicks Building, Hounsfield Road, Sheffield, UK

^3^Centre for Ecology and Conservation, University of Exeter, Cornwall, UK

^4^Département des Sciences Biologiques, Université du Québec à Montréal, Québec, Canada

^5^British Antarctic Survey, Natural Environment Research Council, Cambridge, UK

^6^Centre d'Etudes Biologiques de Chizé, CNRS-La Rochelle Université, Villiers-en-Bois, France

^7^Institute of Alpine and Arctic Research, University of Colorado Boulder, Boulder, CO, USA

^8^Biology Department, Woods Hole Oceanographic Institution, Woods Hole, MA, USA

***Correspondence:** Bertille Mohring, School of Environmental Sciences, University of Liverpool, Liverpool, L693GP UK, [*bmohring@liverpool.ac.uk*](mailto:bmohring@liverpool.ac.uk)

## Supporting Information S1: Robustness test for variation in life-history strategies between populations and individuals using a subset of individual with at least four breeding attempts

To verify the robustness of the results, the analysis presented in 2.2.1. was carried out on a subset of individuals with at least four breeding attempts during the study period (n_Ker_ = 5291, n_ID Ker_ = 548, n_BI_ = 2439, n_ID BI_ = 247), and results remain qualitatively similar.


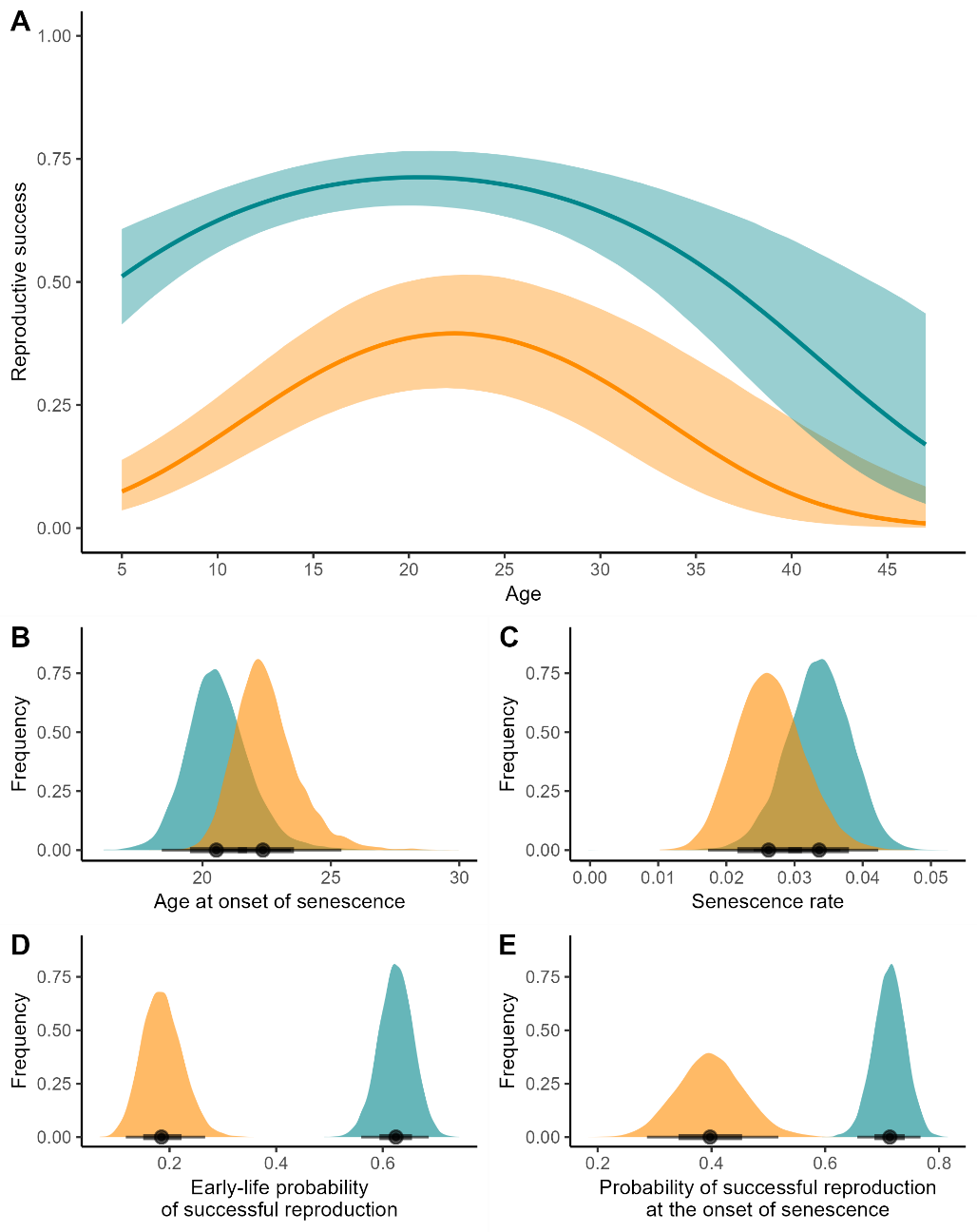


*Figure S1.1: A) Relationship between average population-level reproductive success and age and posterior distribution for each population of predicted (B) age at the onset of senescence, (C) senescence rate, (D) early-life probability of successful reproduction (i.e., at 10yo) and (E) probability of successful reproduction at the onset of senescence in a subset of black-browed albatrosses breeding at Bird Island (in orange) and Kerguelen (in blue) and with at least four breeding attempts during the study period. In (A) lines depict population-level predictions and shaded areas account for 95% confidence interval. In (B-E) areas depict posterior probability distributions.*

Reproductive success increased with age before decreasing at old age in both populations. However, black-browed albatrosses breeding at Kerguelen and Bird Island differed in the shape of their reproductive ageing pattern (Table S1.1, Fig S1.1A). Individuals from Kerguelen displayed higher breeding success than individuals from Bird Island (Table S1.1, Fig S1.1A). Individuals from Kerguelen also displayed a lower linear age slope and a higher quadratic age slope than those from Bird Island (Table S1.1). In addition, individuals displayed a lower probability of successful reproduction when breeding for the first time (Table S1.1). Predicted age at the onset of senescence was higher at Bird Island than at Kerguelen, with an overlap of 42.36 ± 0.49% of posterior distributions (Fig S1.1B). Predicted senescence rates were lower at Bird Island than at Kerguelen, with an overlap of 45.09 ± 0.46% of posterior distributions (Fig S1.1C). Predicted early life and maximum reproductive performances were both higher at Kerguelen than at Bird Island, with little to no overlap between the posterior distributions of the two life-history parameters (predicted probability of successful reproduction at 10 years old: overlap ± SE = 0.00 ± 0.00%, Fig S1.1D; predicted probability of successful reproduction at the onset of senescence: overlap ± SE = 0.13 ± 0.02%, Fig S1.1E).

***Table S1.1****: Parameter estimates (ꞵ) and 95% credible intervals (CI) from the random slope and intercept generalised linear mixed model with a binomial error distribution explaining variation in black-browed albatross reproductive success in response to age (as a linear and quadratic effect), population (Bird Island or Kerguelen) and primiparity or multiparity of the breeding attempt. Individual identity, year and cohort were included as random intercepts linear and quadratic age terms were included as random slopes for each individual. The model was run on a subset of black-browed albatrosses breeding at Bird Island and Kerguelen with at least four breeding attempts during the study period.*

|  | Estimate (ꞵ) | 95% credible intervals (CI) |
| --- | --- | --- |
| Fixed effects |  |  |
| Intercept | -0.65 | (-1.11, -0.19) |
| Age | 0.48 | (0.30, 0.67) |
| Age^2^ | -0.27 | (-0.38, -0.15) |
| Population: Kerguelen | 1.51 | (0.98, 2.04) |
| Age*population: Kerguelen | -0.31 | (-0.52, -0.11) |
| Age^2^*population: Kerguelen | 0.13 | (0.00, 0.26) |
| Breeding attempt: primiparity | -0.50 | (-0.71, -0.30) |
| Random effects |  |  |
| sd of random intercepts on ID: Bird Island | 0.69 | (0.51, 0.90) |
| sd of random intercepts on ID: Kerguelen | 0.57 | (0.43, 0.72) |
| sd of random slopes on age per ID: Bird Island | 0.39 | (0.10, 0.64) |
| sd of random slopes on age per ID: Kerguelen | 0.22 | (0.02, 0.41) |
| sd of random slopes on age^2^ per ID: Bird Island | 0.12 | (0.01, 0.30) |
| sd of random slopes on age^2^ per ID: Kerguelen | 0.14 | (0.01, 0.28) |
| sd of random intercepts on year: Bird Island | 1.21 | (0.90, 1.62) |
| sd of random intercepts on year: Kerguelen | 0.70 | (0.53, 0.91) |
| sd of random intercepts on cohort: Bird Island | 0.18 | (0.01, 0.42) |
| sd of random intercepts on cohort: Kerguelen | 0.10 | (0.00, 0.24) |
| Correlations between random slopes and intercepts |  |  |
| Correlation between random intercept and random age slopes: Bird Island | -0.30 | (-0.77, 0.26) |
| Correlation between random intercept and random age slopes: Kerguelen | -0.00 | (-0.60, 0.55) |
| Correlation between random intercept and random age^2^ slopes: Bird Island | -0.38 | (-0.89, 0.52) |
| Correlation between random intercept and random age^2^ slopes: Kerguelen | -0.44 | (-0.83, 0.30) |
| Correlation between random age and age^2^ slopes: Bird Island | -0.11 | (-0.76, 0.68) |
| Correlation between random age and age^2^ slopes: Kerguelen | -0.28 | (-0.85, 0.59) |

Most life history trait variances were higher at Bird Island than at Kerguelen, the ratio of variance at Bird Island over variance at Kerguelen ranging from 1.02 (predicted senescence rate) to 4.22 (predicted probability of successful reproduction at the onset of senescence; details in Table S1.2). The among-year variance in reproductive success was 3.01 times higher at Bird Island than at Kerguelen (Table S1.2) and the among-cohort variance in reproductive success was 3.21 times higher at Bird Island than at Kerguelen (Table S1.2). After removing two individuals from Kerguelen with little to no senescence, individual predictions of age at the onset of senescence were negatively associated with predictions of senescence rates, both at Bird Island and Kerguelen (correlations, r_BI_ = -0.62; r_Ker_ = -0.60, Fig S1.2).

***Table S1.2****: Among-year and among-cohort variance in reproductive success and among-individual variance in life-history traits (predicted age at onset of senescence, senescence rate, early-life probability of successful reproduction and probability of successful reproduction at the onset of senescence) at Bird Island and Kerguelen, and variance ratio between the two colonies. The model was run on a subset of black-browed albatrosses breeding at Bird Island and Kerguelen with at least four breeding attempts during the study period.*

| Parameter | Bird Island | Kerguelen | Ratio |
| --- | --- | --- | --- |
| Random intercept on year | 1.487 | 0.494 | 3.012 |
| Random intercept on cohort | 0.046 | 0.014 | 3.207 |
| Predicted age at the onset of senescence | 0. 047 | 0. 046 | 1.016 |
| Predicted senescence rate | 0.0010 | 0.0008 | 1.142 |
| Predicted early-life probability of successful reproduction | 0.0014 | 0.0010 | 1.378 |
| Predicted probability of successful reproduction at the onset of senescence | 0.0035 | 0.0008 | 4.220 |


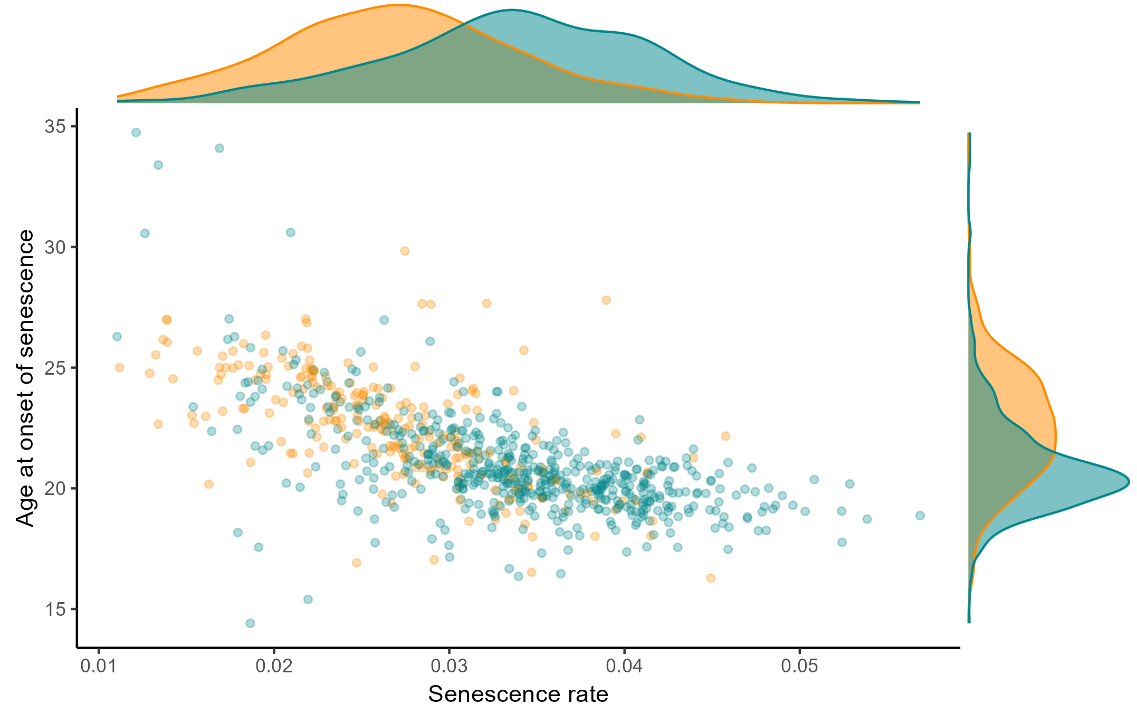


*Figure S1.2: Relationship between individual predicted age at the onset of senescence and senescence rate in black-browed albatrosses breeding at Bird Island (in orange) and Kerguelen (in blue) on a subset of individuals with at least four breeding attempts during the study period.*

Individual lifetime reproductive success was negatively associated with predicted age at the onset of senescence at Bird Island (estimate: -0.13 (-0.26, -0.01), Figs S1.3A,C) and Kerguelen (estimate: -0.22 (-0.29, -0.15), Figs S1.3B,C), with no clear difference in the strength of the relationship between the two populations (interaction term estimate: -0.08 (-0.23, 0.06)). This result indicates that an earlier onset of senescence correlates with higher fitness. Lifetime reproductive success was positively associated with predicted senescence rates both at Bird Island (estimate: 0.52 (0.38, 0.66), Fig S1.3A,D) and Kerguelen (estimate: 0.25 (0.19, 0.31), Fig S1.3B,D), with a stronger relationship between lifetime reproductive success and predicted senescence rates at Bird Island than at Kerguelen (interaction term estimate: 0.28 (0.13, 0.42)). This result suggests stronger senescence rates in individuals producing more offspring during their lifetime.


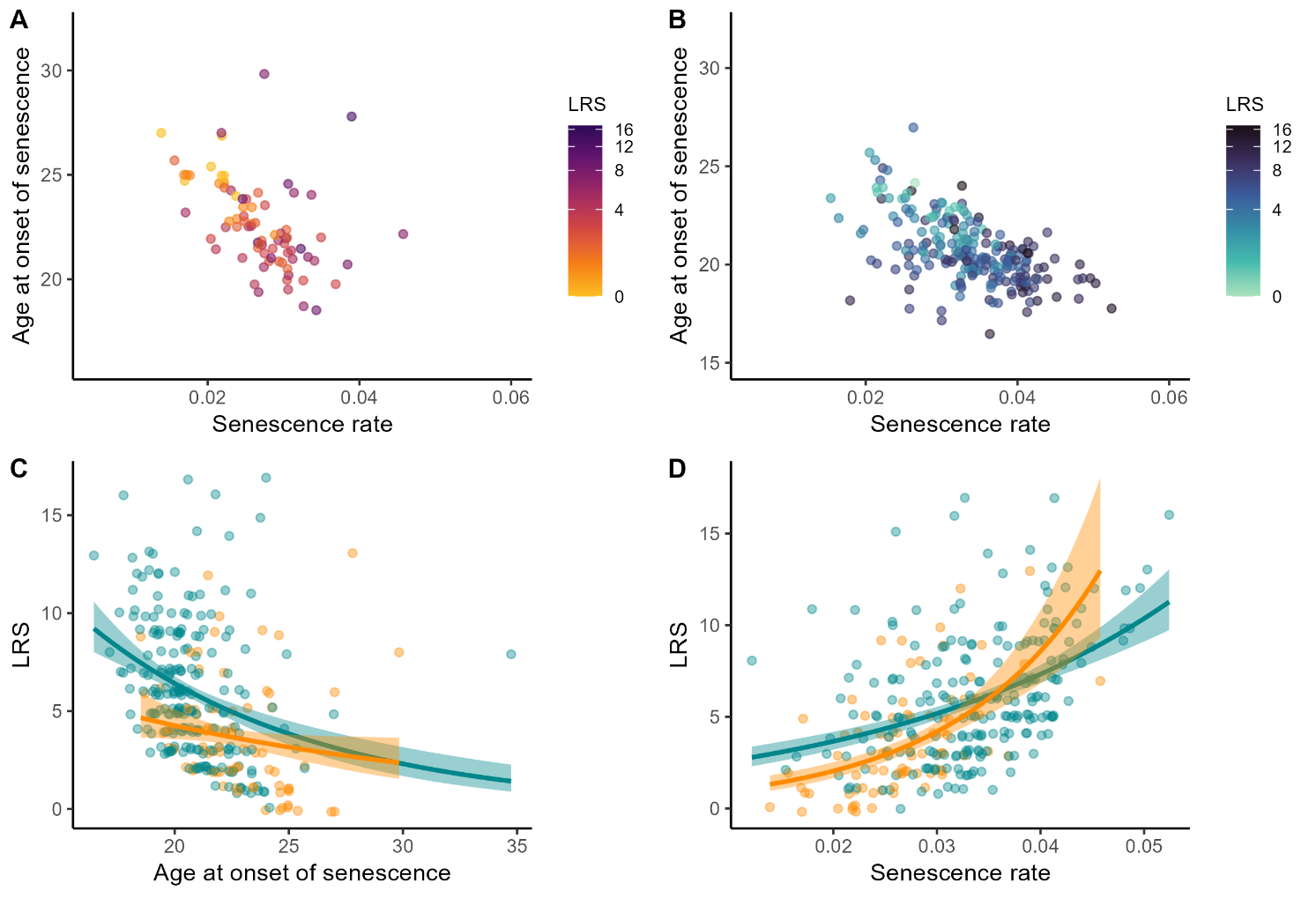


*Figure S1.3: Relationship between individual predicted age at the onset of senescence, predicted senescence rate and lifetime reproductive success (colour gradient) in black-browed albatrosses breeding at (A) Bird Island and (B) Kerguelen fledged before 2000 and presumed dead by the end of the study period. Relationship between lifetime reproductive success and (C) predicted age at the onset of senescence, (D) predicted senescence rate in black-browed albatrosses breeding at Bird Island (in orange) and Kerguelen (in blue) fledged before 2000, and with at least four breeding attempts and presumed dead by the end of the study period (n_ID Ker_ = 219, n_ID BI_ = 80). Lines depict population-level responses and shaded areas account for 95% confidence interval.*

To verify the robustness of the analysis of the association between the individual lifetime reproductive success and predicted age at the onset and rate of senescence, this analysis was also performed on all monitored individuals (with at least four breeding attempts during the study period), including all cohorts and distinguishing individuals presumed dead from individuals that are still alive by the end of the study period (as presented in Supplementary Material S5 for lifetime reproductive success). The estimates of the responses for each combination of population (Bird Island or Kerguelen) and status (presumed dead or still alive) are presented in Table S1.3. Individual lifetime reproductive success was negatively associated with predicted age at the onset of senescence (Table S1.3¸Fig S1.4A) and positively associated with predicted senescence rates (Table S1.3, Fig S1.4B). The results are consistent with those obtained on the subset of individuals fledged before 2000 and presumed dead by the end of the study period.

*Table S1.3: Estimate (95% CI) of the effects of predicted onset of senescence and senescence rate lifetime reproductive success (for birds with at least four breeding attempts during the study period, presumed dead or still alive at the end of the study period). Estimates with 95% CI not overlapping 0 are shown in bold.*

| Population | Bird Island | | Kerguelen | |
| --- | --- | --- | --- | --- |
| Status | Dead | Alive | Dead | Alive |
| n | 96 | 151 | 252 | 294 |
| Predicted onset of senescence | **-0.17 (-0.29, -0.05)** | **-0.33 (-0.42, -0.25)** | **-0.26 (-0.33, -0.19)** | **-0.18 (-0.24, -0.13)** |
| Predicted senescence rate | **0.61 (0.47, 0.76)** | **0.58 (0.49, 0.67)** | **0.28 (0.22, 0.34)** | **0.29 (0.23, 0.36)** |

*
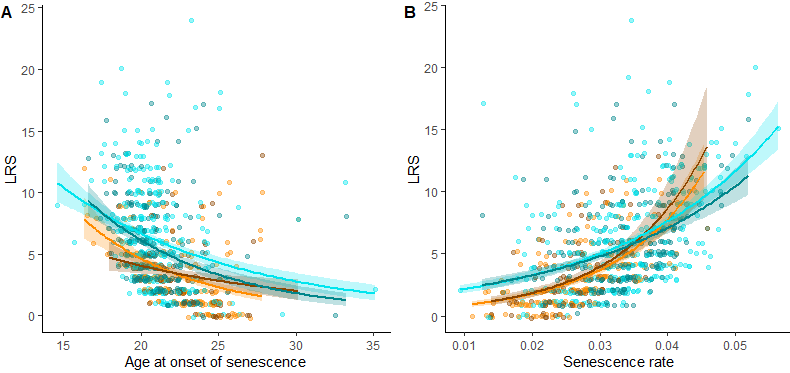
Figure S1.4: Relationship between lifetime reproductive success and (A) predicted age at the onset of senescence, (B) predicted senescence rate in black-browed albatrosses breeding at Bird Island (in orange) and Kerguelen (in blue) on a subset of individuals with at least four breeding attempts during the study period, from all cohorts, either presumed dead (darker shade) or alive (lighter shade) by the end of the study period. Lines depict population-level responses and shaded areas account for 95% confidence interval.*

## Supporting Information S2: Derivation of biologically meaningful metrics of reproductive senescence for each individual

To investigate age-related variation in reproductive success in black-browed albatrosses from Kerguelen and Bird Island populations, we built a generalised linear mixed model with a binomial error distribution:

${RS}_{i,c,y,p} \sim Bernouilli(p_{i,c, y,p})$

$$logit\left( p_{i,c,y,p} \right)= \beta_{0}+\beta_{1}A+\beta_{2}A^{2}+\beta_{3}B+\gamma_{1,p}+\gamma_{2,p}A+\gamma_{3,p}A^{2}+u_{1,p,i}+u_{2,p,i}A+u_{3,p,i}A^{2}+v_{p,y}+w_{p,c}+\epsilon_{i,c,y,p}$$

$u_{k,p,\cdot}\sim N\left( \boldsymbol{0},\Sigma_{k,p}^{u} \right)$, $v_{p,\cdot}\sim N\left( \boldsymbol{0},\Sigma_{p}^{v} \right)$ , $w_{p,\cdot}\sim N\left( \boldsymbol{0},\Sigma_{p}^{w} \right)$ for each $k,p$

Where ${RS}_{i,c,y,p}$ is the reproductive success of the breeding attempt of individual *i* from cohort *c* and population $p$ at age *j* on year *y*; $p_{i,c,y,p}$ is the probability that the breeding attempt of individual $i$ from cohort $c$ at age *j* on year *y* is successful (i.e., that the chick fledges); A is age; B is a Boolean variable controlling for primiparity or multiparity of the breeding attempt; $u_{k,p,\cdot}$ is a vector of all the $u_{k,p,i}$-values for each pair $k, p$; $v_{p,\cdot}$ is a vector of all the $v_{p,y}$-values for each year *y* and population $p$; $w_{p,\cdot}$ is a vector of all the $w_{p,c}$-values for each cohort *c* and population $p$; $u_{k,p,\cdot}$ is a vector of all the $u_{k,p,i}$-values for each population $p$ and individual $i$; and $\Sigma_{k,p}^{u},\Sigma_{p}^{v},\Sigma_{p}^{w}$are covariance matrices for each $p$ and $k$. The individual random effects $u_{k,p,i}$ account for among-individual heterogeneity (in intercept, linear and quadratic age slopes) and for the non-independence of multiple observations of an individual during its lifetime. The random year effect $v_{p,y}$accounts for environmental stochasticity. The random cohort effect $w_{p,c}$ accounts for the non-independence of individuals born on the same year.

To compare black-browed albatross life-history strategies within and among the two populations, we extracted the predicted individual-level values of estimates of intercept, linear and quadratic age effects and we derived biologically meaningful life-history parameters. Specifically, we calculated for each individual: the predicted age at the onset of senescence ($x_{B}$), senescence rate ($r$), individual performance at 10 years old ($y_{A}$) and performance at the age of the onset of senescence ($y_{B}$) (Figure S3.1). The age at onset of senescence was defined as the abscissa at the peak of the curve depicting age-specific variation in reproductive for each individual. For each individual $i$ from cohort $c$, the age at onset of senescence was calculated as $x_{B, i,c}= \frac{- \left( \beta_{1}+ \gamma_{2,p}+ u_{2,p,i} \right)}{2 \left( \beta_{2}+ \gamma_{3,p}+ u_{3,p,i} \right)}$. For each individual $i$ from cohort $c$, senescence rate $r_{i,c}$ was calculated as the slope of the tangent at the inflection point *C* (Figure S3.1). This value was multiplied by $-1$ so that higher values indicate faster senescence and lower values slower senescence.


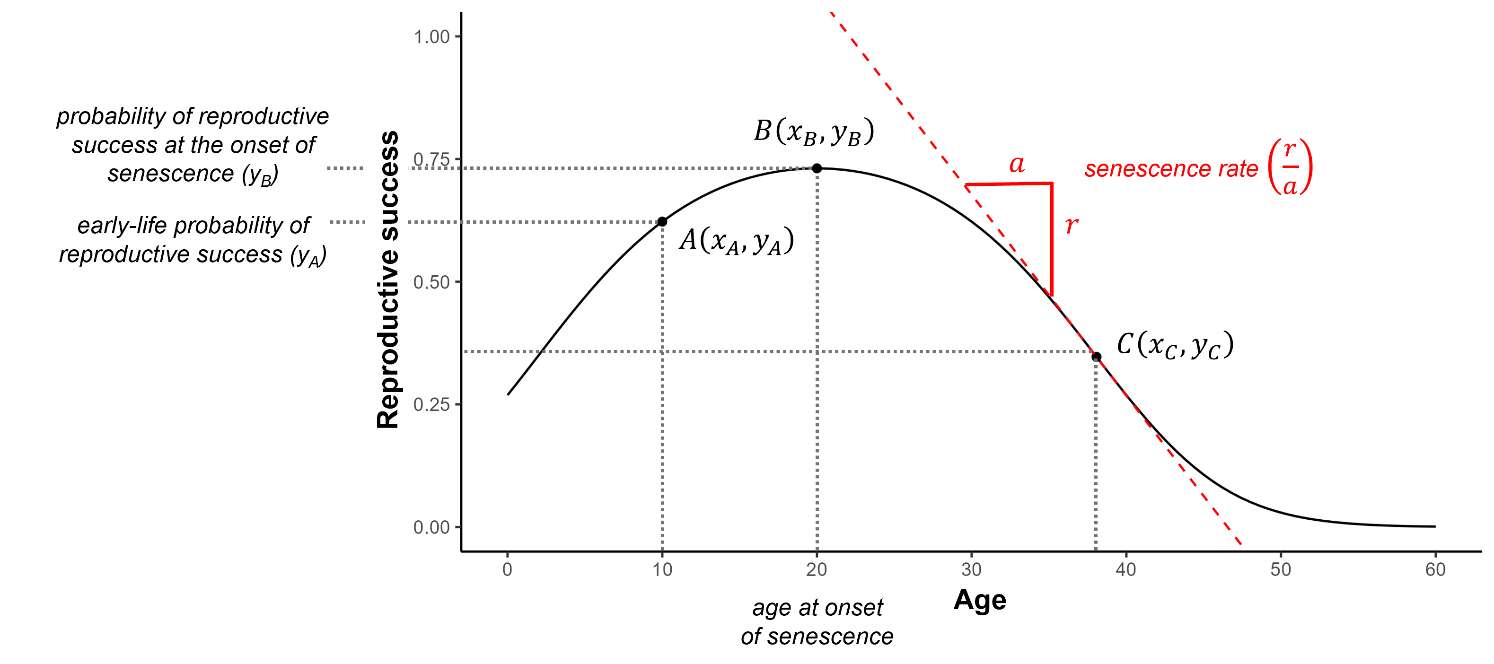


***Figure S2.1****: Curve depicting individual-level variation in the probability of reproductive success with age. Point A is located on the curve at an age of 10 (corresponding to early life in albatrosses). Point B corresponds to the peak of the curve, marking the onset of senescence. Point C represents the first inflection point of the curve, where the rate of change in reproductive success is at its maximum. The dashed red line is the tangent at the inflection point and* $r/a$ *is the senescence rate (i.e., the gradient of the tangent).*

## Supporting Information S3: Relationship between predicted age at onset of senescence and predicted senescence rate when including potential outliers.

When including the individual with little to no reproductive senescence (predicted onset of senescence after 40yo: one individuals), individual predictions of age at the onset of senescence were still negatively associated with predictions of senescence rates, both at Bird Island and Kerguelen (correlations, r_BI_=-0.70; r_Ker_=-0.67, Fig S3.1).


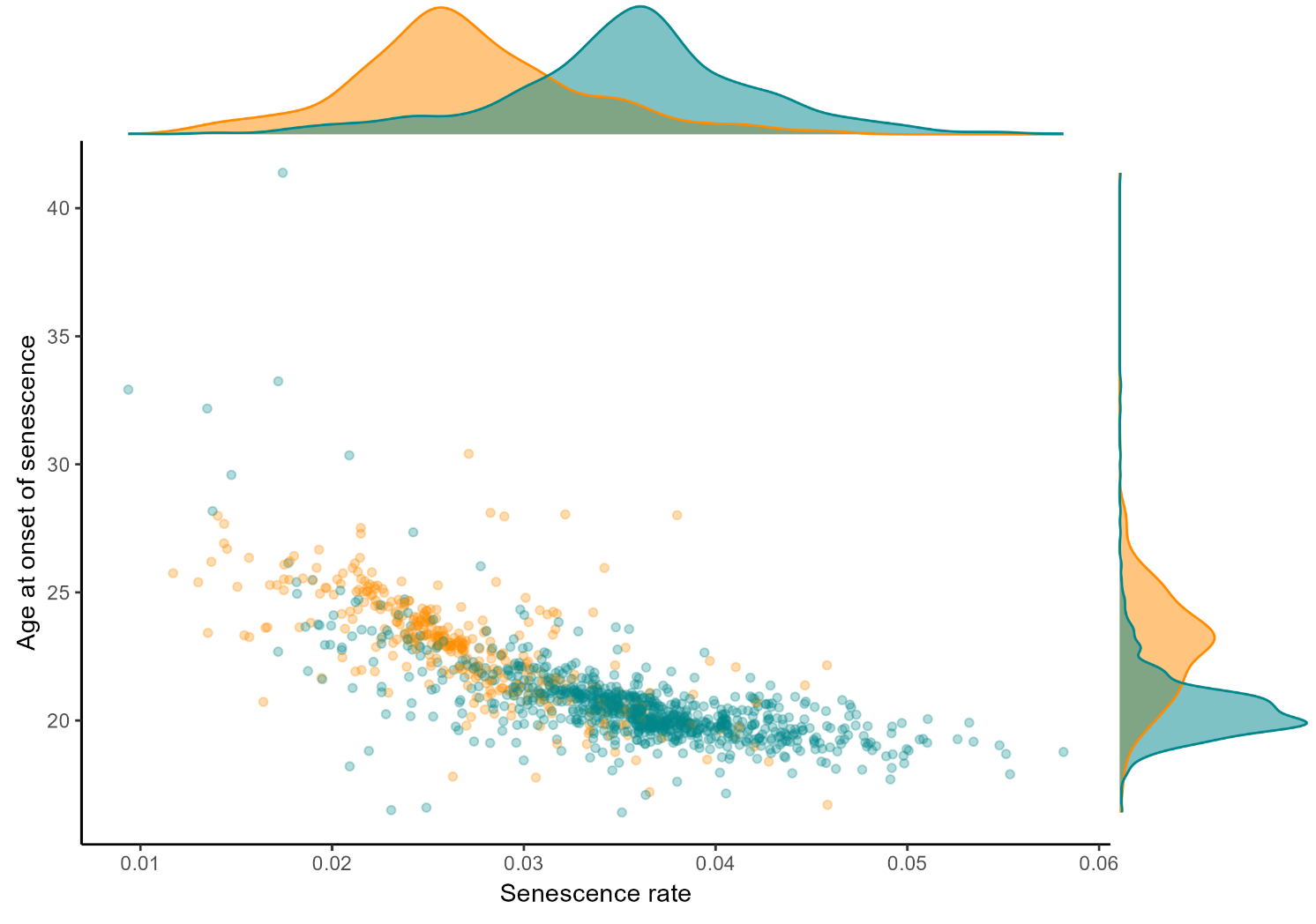


***Figure S3.1****: Relationship between individual predicted age at the onset of senescence and predicted senescence rate in black-browed albatrosses breeding at Bird Island (in orange) and Kerguelen (in blue) when including the individual with no apparent reproductive senescence. Also shown are the density plots of these two variables for each population.*

## Supporting Information S4: Relationship between number of breeding attempts in life, predicted age at onset of senescence and senescence rates.

To assess whether the relationships we observed when investigating the association between fitness and life-history strategies were linked to the costs of successfully fledging a chick or to the cost of attempting breeding, we re-ran the analysis presented in Section 2.2.3 and replaced lifetime reproductive success by the number of breeding attempts in the lifetime. Accordingly, we first built a first generalised linear model (GLM) with a Poisson error distribution with the number of breeding attempts of an individual during its lifetime as the dependent variable and predicted age at the onset of senescence, population and the two-way interaction between population and predicted age at the onset of senescence as independent variables. We then built a GLM with a Poisson error distribution with the number of breeding attempts of an individual during its lifetime as the dependent variable and predicted senescence rate, population and the two-way interaction between population and predicted senescence rate as independent variables.

While we found evidence for a negative relationship between lifetime reproductive success and predicted age at the onset of senescence (see 3.2), our results support a positive relationship between the number of breeding attempts of an individual during its lifetime and predicted age at the onset of senescence at Kerguelen (estimate: 0.10 (0.04, 0.15); Figs S4.1B,C), and a trend for a similar relationship at Bird Island (estimate: 0.06 (-0.01, 0.12); Figs S4.1A,C). We also found a positive relationship between the number of breeding attempts of an individual during its lifetime and predicted senescence rates at Bird Island (estimate: 0.17 (0.07, 0.26); Figs S4.1A,D), and a trend for such a relationship at Kerguelen (estimate: 0.02 (-0.03, 0.07); Figs S4.1B,D). Overall, these results suggest a delayed onset of senescence in birds attempting breeding more during their lifetime, but stronger senescence rates.


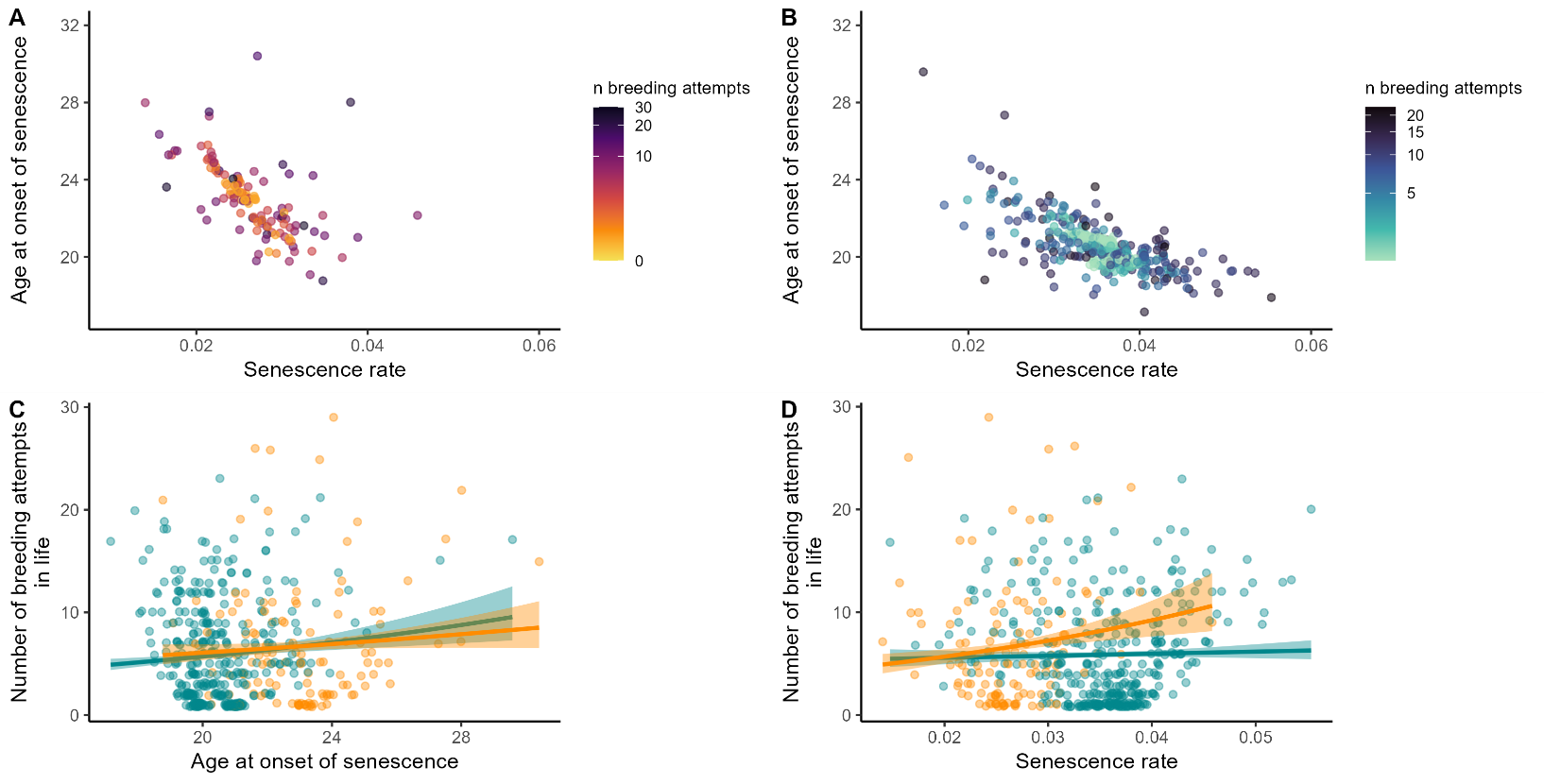


*Figure S4.1: Relationship between the number of breeding attempts during an individual’s lifetime and (A) predicted age at the onset of senescence, (B) predicted senescence rate in black-browed albatrosses breeding at Bird Island (in orange) and Kerguelen (in blue) fledged before 2000 and presumed dead by the end of the study period. Lines depict population-level responses and shaded areas account for 95% confidence interval.*

We further aimed to quantify the variance in the number of breeding attempts or successful breeding attempts of an individual during its lifetime explained by the predicted age at onset of senescence and senescence rate by calculating R^2^ of the different GLMs (‘bayes_R2’ function, brms package; Bürkner, 2017). GLMs including predicted age at onset of senescence explained 1.3% of the variance in the number of breeding attempts of an individual during its lifetime (R^2^ ± SE= 0.013 ± 0.005) and 6.4% of the variance in individual lifetime reproductive success (R^2^ ± SE= 0.064 ± 0.011). Likewise, GLMs including predicted senescence rate explained 1.3% of the variance in the number of breeding attempts of an individual during its lifetime (R^2^ ± SE= 0.013 ± 0.005), whereas they explained 17.4% of the variance in individual lifetime reproductive success (R^2^ ± SE= 0.174 ± 0.021). Overall, these results suggest that the onset and rate of senescence may play a stronger role in explaining variation in the number of offsprings produced by an individual during its lifetime than in the number of breeding attempts. Life-history strategies may therefore be more linked to the cost successfully fledging a chick than to the cost of attempting breeding.

To verify the robustness of the analysis of the association between the number of breeding attempts of an individual during its lifetime and predicted age at the onset and rate of senescence, this analysis was also performed on all monitored individuals, including all cohorts and distinguishing individuals presumed dead from individuals that are still alive by the end of the study period (as presented in Supplementary Material S6 for lifetime reproductive success). The estimates of the responses for each combination of population (Bird Island or Kerguelen) and status (presumed dead or still alive) are presented in Table S4.1.

As for the subset of dead birds fledged before 2000, we found a positive relationship between the number of breeding attempts during an individual’s lifetime and predicted age at onset of senescence (Table S4.1, Fig S4.2A). This relationship was evident for both populations and both status, although CI slightly overlapped 0 at Bird Island (Table S4.1). Accordingly, a higher number of breeding attempts was associated with a later onset of senescence. We also found a positive relationship between the number of breeding attempts during an individual’s lifetime and predicted senescence rates (with some CI slightly overlapping 0; Table S4.1, Fig S4.2 B). These results suggested stronger senescence rates in birds attempting breeding more during their lifetime. Results are therefore consistent when considering the entire dataset instead of the subset of presumed dead birds.

*Table S4.1: Estimate (95% CI) of the effects of predicted onset of senescence and senescence rate on the number of breeding attempts during an individual’s lifetime (for birds presumed dead or still alive at the end of the study period). Estimates with 95% CI not overlapping 0 are shown in bold.*

| Population | Bird Island | | Kerguelen | |
| --- | --- | --- | --- | --- |
| Status | Dead | Alive | Dead | Alive |
| n | 161 | 199 | 470 | 403 |
| Predicted onset of senescence | 0.06 (-0.00, 0.13) | 0.04 (-0.00, 0.08) | **0.10 (0.04, 0.15)** | **0.05 (0.02, 0.09)** |
| Predicted senescence rate | **0.19 (0.09, 0.29)** | 0.04 (-0.01, 0.10) | 0.04 (-0.02, 0.09) | **0.06 (0.03, 0.10)** |

**
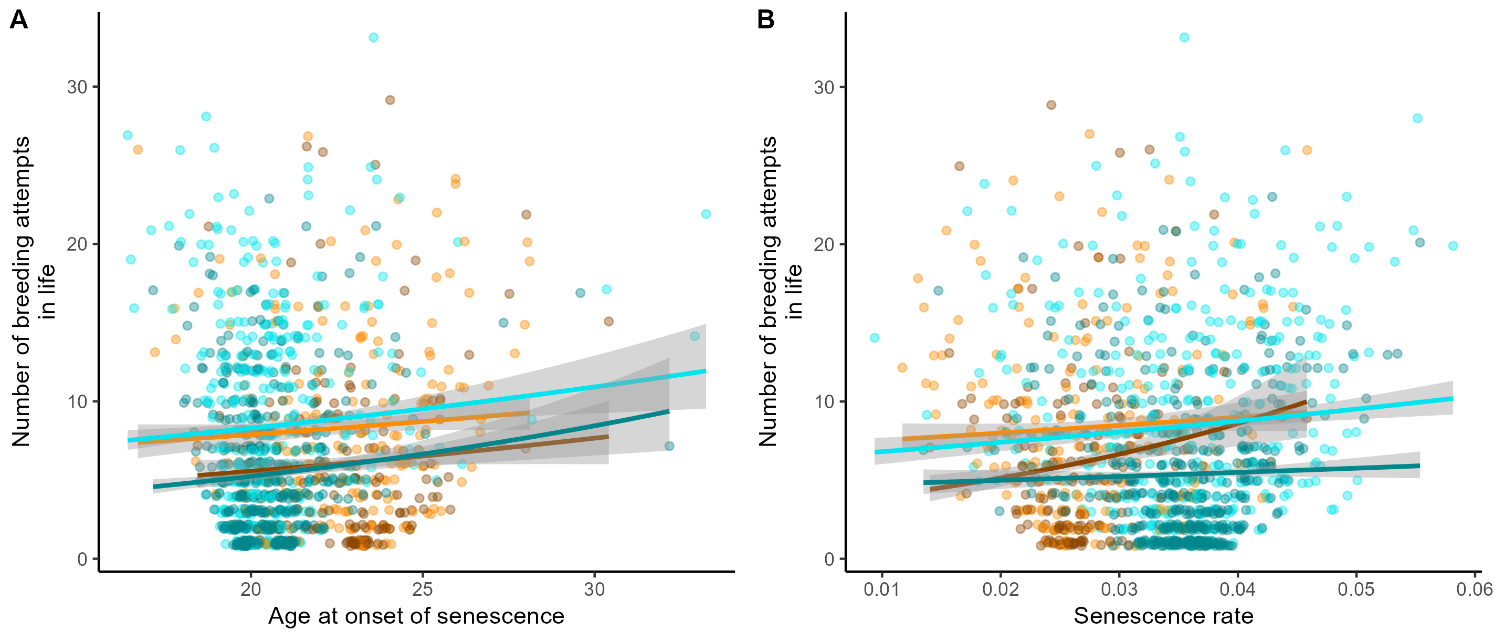
**

*Figure S4.2: Relationship between the number of breeding attempts during an individual’s lifetime and (A) predicted age at the onset of senescence, (B) predicted senescence rate in black-browed albatrosses breeding at Bird Island (in orange) and Kerguelen (in blue) from all cohorts, either presumed dead (darker shade) or alive (lighter shade) by the end of the study period. Lines depict population-level responses and shaded areas account for 95% confidence interval.*

## Supporting Information S5: Robustness test for the fitness outcomes of the life-history strategies exhibited by black-browed albatrosses, using all available data

To ensure that the results obtained when investigating the relationship between fitness and individual life-history strategy were not biased towards birds with a short lifespan – possibly due to external mortality causes such as fisheries bycatch rather than ageing – we carried out the analyses presented in 2.2.3. on the entire dataset. In this dataset, we distinguished birds presumed dead (hereafter, dead) from birds that were still alive at the end of the study period (hereafter, alive). Noteworthy, lifetime reproductive success is likely underestimated for the alive subset of individuals, as these birds may have several opportunities to breed in the future.

In the GLMs investigating the relationship between fitness (lifetime reproductive success, as the dependent variable) and independent variables of interest (predicted age at onset of senescence and predicted senescence rate), we replaced the two-way interactions between aforementioned variables and population by three-way interactions between aforementioned variables, population and individual status (dead or alive). We report the estimates of the responses for each combination of population and status in Table S5.1.

Overall, the results were qualitatively similar to the ones presented in 3.2. We found a negative association between lifetime reproductive success and predicted age at onset of senescence (Table S5.1, Fig S5.1 A). This association was found for all populations and status. For both populations and both status, we found a positive association between lifetime reproductive success and predicted senescence rate (Table S5.1, Fig S5.1 B).

*Table S5.1: Estimate (95% CI) of the effects of predicted onset of senescence and senescence rate on lifetime reproductive success for birds presumed dead or still alive at the end of the study period. Estimates with 95% CI not overlapping 0 are shown in bold.*

| Population | Bird Island | | Kerguelen | |
| --- | --- | --- | --- | --- |
| Status | Dead | Alive | Dead | Alive |
| n | 161 | 199 | 468 | 400 |
| Predicted onset of senescence | **-0.28 (-0.41, -0.17)** | **-0.36 (-0.43, -0.28)** | **-0.36 (-0.45, -0.27)** | **-0.18 (-0.24, -0.12)** |
| Predicted senescence rate | **0.84 (0.70, 0.97)** | **0.66 (0.57, 0.74)** | **0.44 (0.37, 0.50)** | **0.38 (0.34, 0.43)** |

*
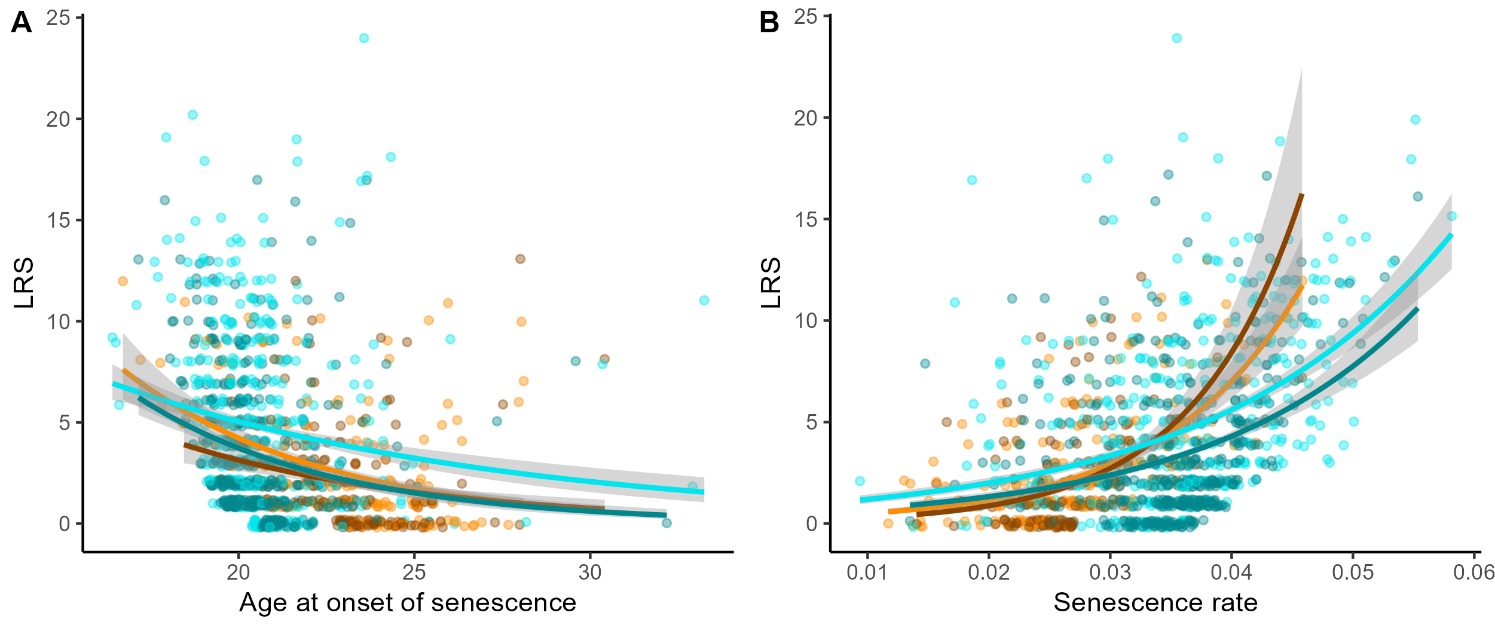
*

*Figure S5.1: Relationship between lifetime reproductive success (LRS) and (A) predicted age at the onset of senescence, (B) predicted senescence rate in black-browed albatrosses breeding at Bird Island (in orange) and Kerguelen (in blue) from all cohorts, either presumed dead (darker shade) or alive (lighter shade) by the end of the study period. Lines depict population-level responses and shaded areas account for 95% confidence interval.*

## Supporting Information S6: Propagation of uncertainty in the estimation of age at the onset of senescence and senescence rate

We tested the robustness of the GLMs linking individual lifetime reproductive success with the predicted age at the onset of senescence and the rate of senescence by propagating the uncertainty associated with the estimation of individual-level age at the onset of senescence and senescence rate. To do so, we randomly selected 2000 iterations of the GLMM described in 2.2.1 (testing for variation in reproductive performance with age), drawn from the 95% credible intervals of age at the onset of senescence and senescence rate. For each iteration, we estimated individual-level values for age at the onset of senescence and senescence rate (as detailed in 2.2.2). For each of the 2000 iterations, we re-ran the two GLMs linking lifetime reproductive success and estimated age at the onset of senescence and senescence rate (detailed in 2.2.3). Potential outliers were excluded, defined as individuals predicted to show little or no senescence (predicted age at the onset of senescence above 40 years old or below 0 years old, or negative predicted senescence rate). We thus obtained 2000 estimates of the relationship between lifetime reproductive success and age at the onset of senescence (respectively lifetime reproductive success and senescence rate), from which we calculated average values and 95% credible interval of the slopes. Although 95% credible intervals overlap zero, the direction of the effects remain consistent. Accordingly, our results supported a negative association between lifetime reproductive success and age at onset of senescence (average (95% CI): Bird Island: -0.08 (-0.34, 0.15), Kerguelen: -0.04 (-0.17, 0.09), Figure S7.1) and a positive association between lifetime reproductive success and senescence rate (average (95% CI): Bird Island: 0.21 (-0.05, 0.61), Kerguelen: 0.10 (-0.04, 0.26), Figure S7.1).


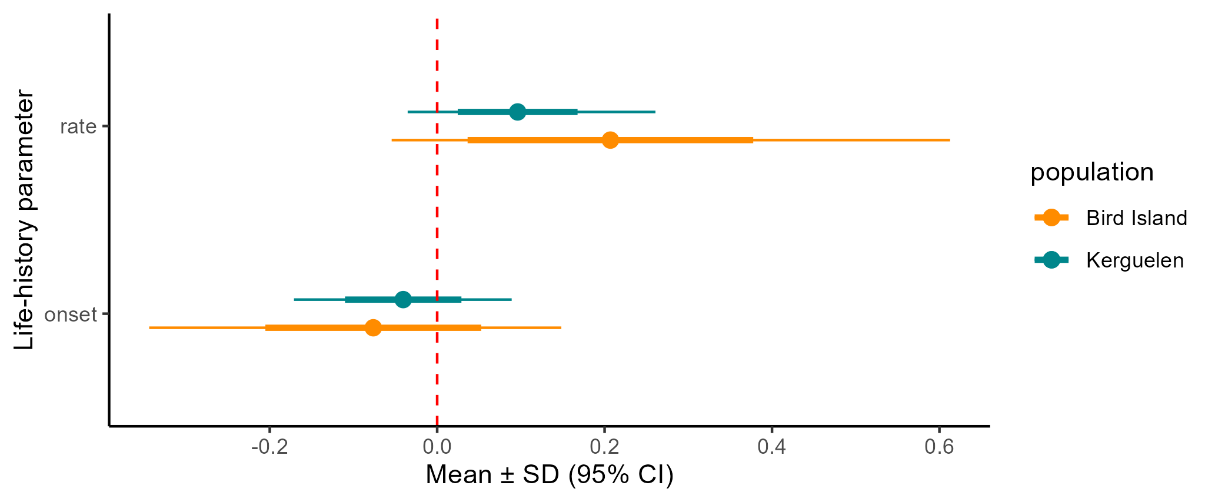


*Figure S6.1: Robustness analysis of the association between lifetime reproductive success and predicted age at onset of senescence (“onset”) and senescence rate (“rate”) for black-browed albatrosses breeding at Bird Island (orange) and Kerguelen (blue). Points represent mean parameter estimate, with error bars showing the standard deviation (thick lines) and 95% credible interval (thin lines). The dashed vertical red line indicates zero.*

## Supporting Information S7: Correlation between life-history parameters


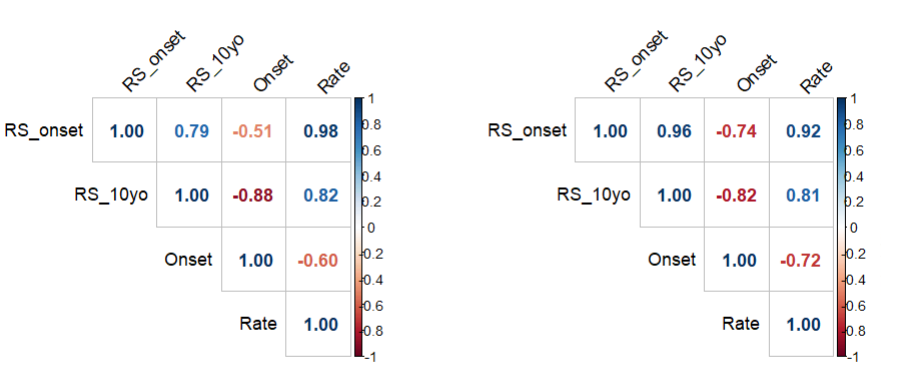


*Figure S7.1: Correlation between life-history parameters for black-browed albatrosses breeding at (A) Bird Island and (B) Kerguelen fledged before 2000 and presumed dead by the end of the study period.*

*‘Onset’:age at onset of senescence; ‘Rate’: senescence rate; ‘RS_10yo’: early-life probability of successful reproduction and ‘RS_onset’: probability of successful reproduction at the onset of senescence.*
